# Computational framework for multi-objective optimization of activated biochar properties using machine learning and evolutionary algorithms

**DOI:** 10.1038/s41598-026-50569-0

**Published:** 2026-05-18

**Authors:** Mohammad Fazle Rabbi

**Affiliations:** Coordination and Research Centre for Social Sciences, Faculty of Economics and Business, University of Debrecen, Böszörményi út 138, Debrecen, 4032 Hungary

**Keywords:** Biochar optimization, Machine learning, Carbon sequestration, Electrochemical energy storage, Multi-objective optimization, Chemistry, Energy science and technology, Engineering, Environmental sciences

## Abstract

Climate neutrality and renewable energy expansion demand multifunctional materials capable of simultaneous carbon sequestration and electrochemical energy storage. Agricultural residue biochar offers dual-function potential, yet conventional pyrolysis does not systematically optimize competing objectives. This study presents a simulation-based computational framework integrating multi-output random forest surrogate modeling with differential evolution algorithms to identify Pareto-optimal process configurations balancing specific surface area, CO_2_ adsorption, electrochemical capacitance, and carbon stability. 800 parameter combinations were evaluated across pyrolysis temperature (400–900 °C), residence time (0.5–3.0 h), heating rate (5–50 °C min^− 1^), activation chemistry (KOH, $\:{\mathrm{H}}_{3}{\mathrm{PO}}_{4}$, $\:{\mathrm{ZnCl}}_{2}$, NaOH), and five feedstock classes using calibrated response surfaces. The surrogate model achieved test-set $\:{\mathrm{R}}^{2}$ of 0.971 (RMSE = 48.06 m^2^ g^− 1^ for specific surface area and 0.942 (RMSE = 6.67 F g^− 1^ for specific capacitance on 160 independent samples; CO_2_ adsorption yielded $\:{\mathrm{R}}^{2}$ = 0.788, while carbon stability index showed moderate fidelity ($\:{\mathrm{R}}^{2}$ = 0.497, RMSE = 0.069), consistent with challenges in modeling recalcitrance from compositional proxies. Multi-objective optimization identified configurations with specific surface area of 1094 m^2^ g^− 1^, $\:{\mathrm{CO}}_{2}$ adsorption of 5.01 mmol g^− 1^, and specific capacitance of 114 F g^−1^. Pyrolysis temperature was the dominant predictor (48% feature importance); hydrogen-to-carbon ratios below 0.4 demarcate recalcitrant materials suitable for millennial-scale sequestration. Optimized processing achieved feedstock-independent carbon stability (median 0.44–0.46) across all biomass types. Spatially explicit assessment indicates that EU-scale deployment could sequester 53.9 Mt$\:{\mathrm{CO}}_{2}$e year^−1^, equivalent to 1.2% of total EU greenhouse gas emissions. Experimental validation of selected configurations constitutes the primary direction for future work.

**Supplementary Information:**

The online version contains supplementary material available at 10.1038/s41598-026-50569-0.

## Introduction

Global efforts seek to constrain atmospheric carbon dioxide concentrations below 450 ppm^1,2^. This threshold is associated with limiting warming to 1.5 °C above preindustrial levels and increasingly relies on negative emission technologies capable of removing and durably sequestering gigatonnes of carbon annually^3,4^. Biochar deployment represents a promising pathway, with a global technical mitigation potential exceeding 2.6 GtCO₂e per year^5^. This potential is realized when agricultural and forestry residues are converted via optimized pyrolysis and integrated into soils or construction materials^2,6^. Concurrently, the accelerating transition toward renewable energy infrastructure has intensified demand for sustainable electrochemical storage systems, particularly supercapacitors offering high power density, rapid charge- discharge cycles, and extended operational lifespans^7,8^. Activated carbons derived from biomass offer a sustainable alternative to fossil-derived electrode materials, yet conventional production methods rarely optimize simultaneously for carbon stability and electrochemical performance^9,10^. Current biochar research has incompletely resolved the design challenge of simultaneously optimizing millennial-scale sequestration and competitive energy storage metrics^11,12^. This unresolved dual function limits the materials contribution to integrated climate and energy strategies.

Agricultural residue valorisation through pyrolysis operates within circular economy frameworks, yet current protocols yield inconsistent material properties due to complex interactions among pyrolysis temperature, residence time, heating rate, activation agent, and feedstock composition^13,14^. The resulting performance heterogeneity reflects the absence of systematic optimization frameworks, and the proliferation of non-standardized experimental datasets impedes cross-study synthesis of generalizable process-property relationships needed for scaling beyond laboratory conditions^15,16^. Pyrolysis temperature exerts dominant control over structural and chemical properties through progressive dehydrogenation, aromatization, and micropore development^17,18^. Alkali activation with potassium hydroxide enhances specific surface area through carbothermal etching, yielding hierarchical pore networks that facilitate both gas adsorption and ion transport^19^. Positive correlations between surface area and electrochemical capacitance depend on electrolyte chemistry and pore accessibility^20,21^, while carbon stability, assessed through atomic $\:\mathrm{H/C}$ and $\:\mathrm{O/C}$ ratios, exhibits inverse relationships with electrochemical performance because aggressive carbonization conditions that maximize aromaticity often collapse mesopores essential for ion diffusion^22^.

Machine learning applications employing random forests, support vector machines, and artificial neural networks have demonstrated varying success for structural descriptors yet show limited capability in capturing stability indices or composite performance metrics^23–27^. Multi-objective optimization studies utilizing genetic algorithms or particle swarm methods have identified Pareto frontiers, though most implementations remain confined to narrow parameter ranges or single feedstock systems^25,26,28,29^. Critical gaps persist in translating process-level optimization into spatially explicit climate assessments, as existing studies focus on material-scale performance without integrating regional biomass availability, logistical constraints, or lifecycle energy balances^30,31^. Standardized surrogate modelling frameworks capable of predicting multiple correlated properties simultaneously, including surface area, CO_2_ adsorption, capacitance, stability, and process energy, are absent^32–34^; the thermochemical mechanisms governing the trade-off between hydrogen-rich functionalities and aromatic condensation remain inadequately quantified with respect to compositional thresholds delineating labile from sequestration-grade materials^35,36^. The scarcity of integrated frameworks coupling machine learning prediction, evolutionary optimization, and regional deployment modelling represents the methodological gap this study addresses.

Three critical limitations characterize existing computational approaches to biochar optimization. First, most studies employ single-output predictive models rather than correlated multi-property vectors. Sun et al. (2024) demonstrated machine learning prediction of capacitance characteristics but limited analysis to single electrochemical properties^23^, while Jiang et al. (2025) focused exclusively on specific surface area prediction without integrating stability or functional performance metrics^37^. Zhang et al. (2025) achieved R²=0.94 for biochar stability prediction yet did not simultaneously model electrochemical or adsorptive properties within a unified framework^35^. This single-property focus prevents systematic evaluation of trade-offs among competing design objectives that govern dual-function material performance. Second, implementations remain confined to narrow parameter ranges or single feedstock systems, restricting generalizability. Song et al. (2025) examined fast versus slow pyrolysis of alkali lignin but limited analysis to a single feedstock and two thermal regimes^18^, while Tiwari and Chinthala (2025) compared conventional and microwave pyrolysis exclusively for tea waste biochar^33^. Wang et al. (2024) investigated coke formation on biochar catalysts but did not extend findings across diverse biomass portfolios or activation chemistries^17^. This narrow parametric scope limits identification of generalizable process-property relationships applicable across regional biomass resources. Third, frameworks rarely integrate material-scale predictions with regional deployment assessments accounting for biomass availability and net energy balances^16,30,31,38^. To address these limitations, this study presents a simulation-based computational framework integrating multi-output random forest surrogate modelling with differential evolution algorithms to identify Pareto-optimal process configurations.

The present framework addresses the three limitations identified above through a methodological integration that is absent from prior work in each area individually. Existing multioutput prediction studies in materials design have not coupled ensemble surrogates with evolutionary multiobjective optimization within a single computational pipeline; existing biochar optimization studies employing genetic or particle swarm algorithms have operated on narrow parameter ranges or single feedstock systems without extending to regionally differentiated deployment assessment; and existing regional biochar deployment models have not been driven by computationally optimized process parameters. The present study combines these three elements within one reproducible framework, and it is this integration rather than any individual component that constitutes the methodological contribution.

This study pursues three interrelated research objectives. The first is to assess the degree to which multioutput surrogate-guided optimization can simultaneously improve structural, electrochemical, and stability performance relative to unoptimized baseline configurations spanning the same parameter space; the working hypothesis is that Pareto-optimal process configurations will yield measurable simultaneous gains across competing property targets rather than improving one at the expense of others. The second is to characterize the compositional thresholds, particularly hydrogen-to-carbon and oxygen-to-carbon atomic ratios, that govern the transition between labile and recalcitrant carbon across diverse thermal regimes and feedstock classes; the working hypothesis is that thermal processing conditions exert a dominant and feedstock-independent control over carbon recalcitrance through progressive aromatization. The third is to estimate the spatially differentiated biochar deployment potential across European agricultural regions when process parameters are set to computationally optimized values; the working hypothesis is that regional differences in residue availability produce heterogeneous total mitigation capacities while mitigation intensity per unit of processed residue remains relatively stable across regions. Together, these objectives advance the broader aim of developing simulation-based computational frameworks that can systematically navigate trade-offs in materials design prior to experimental campaign design.

The study contributes at three levels. Methodologically, it demonstrates that multioutput ensemble learning combined with differential evolution optimization can navigate competing thermochemical objectives in a high-dimensional process parameter space spanning diverse feedstock classes and activation chemistries. Theoretically, it provides a quantitative analysis of compositional indicators as proxies for long-term carbon recalcitrance, contributing to ongoing efforts to establish reliable stability metrics applicable across heterogeneous biomass sources. From an applied perspective, it connects process-level optimization to a spatially explicit regional assessment, providing a structured example of how computational materials design can inform deployment-scale climate mitigation planning. Experimental validation of the identified optimal configurations constitutes the necessary next step beyond the present computational scope.

## Methods and methodology

The study adopts a fully computational design in which activated biochar production is represented through a parametric process space, an ensemble surrogate model, and a multiobjective optimization layer. This approach was chosen because exhaustive experimental coverage of the parameter combinations examined here is prohibitively costly and because the primary objective is to develop and validate the optimization methodology rather than to characterize specific synthesized materials. A simulation-based dataset of approximately 800 parameter combinations was generated from empirically calibrated response surfaces constrained to literature-reported property bounds, providing a controlled environment in which surrogate model accuracy and optimization behavior can be assessed without confounding from experimental measurement noise or feedstock batch variability. The surrogate model maps the seven-dimensional process input vector to six correlated biochar properties simultaneously, and a differential evolution algorithm searches the resulting response surface to identify Pareto-optimal configurations balancing structural, electrochemical, stability, and energetic objectives. The methods are described in the order of their logical dependence; dataset construction and variable definitions precede model specification, which precedes optimization formulation and regional deployment assessment. All computations were implemented in Python 3.14 with fixed random seeds to ensure deterministic reproducibility across all stochastic operations.

### Study design and analytical framework

The study followed a simulation based and fully computational design in which activated biochar production was represented by a parametric process space, a machine learning surrogate, and a multi objective optimization layer.

#### Terminology clarification

This study distinguishes between “machine learning” and “artificial intelligence” for conceptual precision. The term “machine learning” refers specifically to the random forest ensemble modeling approach (Sect.  2.6) implementing supervised learning for property prediction. The term “artificial intelligence” denotes the integrated computational framework combining machine learning surrogates with evolutionary optimization algorithms (Sect.  2.8) to solve multi-objective design problems. Where both terms apply, “machine learning” is preferred to reflect the specific algorithmic implementation.

The decision vector for any candidate configuration is

1 $$\:\begin{array}{c}x=[{T}_{\mathrm{pyr}},\:{t}_{\mathrm{res}},\:{r}_{\mathrm{heat}},\:{r}_{\mathrm{act}},\:f,\:a,\:R{]}^{\mathsf{T}}\end{array}$$

where $\:{T}_{\mathrm{pyr}}$ is pyrolysis temperature (°C), $\:{t}_{\mathrm{res}}$ residence time (h), $\:{r}_{\mathrm{heat}}$ heating rate (°C,$\:{\mathrm{m}\mathrm{i}\mathrm{n}}^{-1}$), $\:{r}_{\mathrm{act}}$ activation agent‑to‑char mass ratio, $\:f$ feedstock type, $\:a$ activation agent, and $\:R$ deployment region. The surrogate model implements a deterministic mapping

2 $$\:\begin{array}{c}y=f\left(x\right)=[\mathrm{S}\mathrm{S}\mathrm{A},\:{q}_{{\mathrm{CO}}_{2}},\:{C}_{\mathrm{spec}},\:{E}_{\mathrm{cap}},\:{S}_{\mathrm{C}},\:{E}_{\mathrm{proc}}{]}^{\mathsf{T}}\end{array}$$

where SSA is specific surface area (m² $\:{\mathrm{g}}^{-1}$), $\:{q}_{{\mathrm{CO}}_{2}}$ CO_2_ adsorption capacity (mmol $\:{\mathrm{g}}^{-1}$), $\:{C}_{\mathrm{spec}}$ specific capacitance (F $\:{\mathrm{g}}^{-1}$), $\:{E}_{\mathrm{cap}}$ energy storage capacity (Wh $\:\mathrm{k}{\mathrm{g}}^{-1}$), $\:{S}_{\mathrm{C}}$ carbon stability index (–), and $\:{E}_{\mathrm{proc}}$ process energy demand (MJ $\:\mathrm{k}{\mathrm{g}}^{-1}$). The optimisation problem is thus defined in the joint space of $\:x$ and $\:y$, with structural, electrochemical, stability, and energetic criteria evaluated simultaneously.

### Data sources, simulation setup, and database integration

This section describes the construction of the computational dataset used for surrogate model training and validation. The approach integrates empirically calibrated response surfaces with systematic parameter space sampling to generate a synthetic database representing realistic biochar property ranges reported in experimental literature. Data preprocessing, partitioning protocols, and quality assurance procedures ensuring physical consistency are detailed below.

#### Simulation based dataset construction

To enable systematic exploration of the high-dimensional parameter space encompassing seven input variables and six output properties, a computational dataset was generated using empirically calibrated response surfaces rather than experimental synthesis. This design decision was adopted because exhaustive experimental coverage of the parameter combinations defined in Eqs. 3 and 4 is prohibitively costly, because simulation allows standardized property metrics across heterogeneous literature conditions, and because the primary objective of the study is to validate the optimization methodology itself rather than to characterize specific synthesized materials. Experimental confirmation of selected configurations constitutes necessary future work.

Response surface functions $\:\mu\:\left(x\right)$ for each of the six output properties (Eq. 7 through 12) were parameterized through a three-step calibration procedure. In the first step, property ranges were extracted from 45 peer-reviewed experimental biochar studies published between 2015 and 2025, spanning diverse feedstocks (wood, agricultural residues, sewage sludge), thermal conditions ($\:{T}_{\mathrm{pyr}}$ = 300 to 900 °C), and activation protocols (KOH, H_3_PO_4_, ZnCl_2_, NaOH). This survey established the following feasible bounds; specific surface area from 50 to 1500 m^2^ g^− 1^; specific capacitance from 50 to 200 F g^− 1^; CO_2_ adsorption from 0.5 to 6.0 mmol g^− 1^; carbon stability index from 0.2 to 0.8; process energy from 0.8 to 10.0 MJ kg^− 1^. In the second step, the polynomial and logarithmic functional forms in Eq. 7 through 12 were selected to reproduce the nonlinear relationships reported in the surveyed studies, including the logarithmic saturation of CO_2_ adsorption capacity with increasing surface area observed above 1200 m^2^ g^− 1^. In the third step, regression coefficients ($\:{\beta\:}_{0}$ through $\:{\beta\:}_{3}$ in Eq. 7; $\:{\gamma\:}_{0}$, $\:{\gamma\:}_{1}$ in Eq. 8; $\:{\delta\:}_{0}$ through $\:{\delta\:}_{2}$ in Eq. 9; $\:{\theta\:}_{1}$, $\:{\theta\:}_{2}$ in Eq. 11) were fitted by minimizing least-squares residuals between $\:\mu\:\left(\mathbf{x}\right)$ predictions and median property values extracted from the literature survey. Gaussian noise $\:\epsilon\:\sim\:\mathcal{N}(0,{\Sigma\:})$ with standard deviations proportional to 5 to 10% of property means was subsequently superimposed per Eq. 5 to represent residual experimental variability. The resulting dataset constitutes an empirically constrained parametric construction rather than a mechanistic first-principles simulation; the surrogate model trained on this dataset therefore functions as an interpolation instrument within the calibrated property space and is not applied beyond the literature-validated bounds defined above.

#### Parameter space definition

Continuous process variables were sampled from bounded distributions

3 $$\:\begin{array}{c}{T}_{\mathrm{pyr}}\in\:\left[\mathrm{400,900}\right],\:\:{t}_{\mathrm{res}}\in\:\left[\mathrm{0.5,4.0}\right],\:\:{r}_{\mathrm{heat}}\in\:\left[\mathrm{5,50}\right],\:\:{r}_{\mathrm{act}}\in\:\left[\mathrm{0.5,3.0}\right]\end{array}$$

where $\:{T}_{\mathrm{pyr}}$ is pyrolysis temperature (°C), $\:{t}_{\mathrm{res}}$ residence time (h), $\:{r}_{\mathrm{heat}}$ heating rate (°C min^− 1^), and $\:{r}_{\mathrm{act}}$ activation agent to char mass ratio. Discrete factors were drawn from

4 $$\:\begin{array}{c}f\in\:\left\{\mathrm{wood},\:\mathrm{sludge},\:\mathrm{coconut},\:\mathrm{straw},\:\mathrm{rice\:husk}\right\},\:\\\:a\in\:\left\{\mathrm{KOH},\:\mathrm{NaOH},\:{\mathrm{ZnCl}}_{2},\:{\mathrm{H}}_{3}{\mathrm{PO}}_{4}\right\},\:\\\:R\in\:\{\mathrm{E}\mathrm{a}\mathrm{s}\mathrm{t}\mathrm{e}\mathrm{r}\mathrm{n},\mathrm{C}\mathrm{e}\mathrm{n}\mathrm{t}\mathrm{r}\mathrm{a}\mathrm{l},\mathrm{N}\mathrm{o}\mathrm{r}\mathrm{t}\mathrm{h}\mathrm{e}\mathrm{r}\mathrm{n},\mathrm{S}\mathrm{o}\mathrm{u}\mathrm{t}\mathrm{h}\mathrm{e}\mathrm{r}\mathrm{n},\mathrm{W}\mathrm{e}\mathrm{s}\mathrm{t}\mathrm{e}\mathrm{r}\mathrm{n}\}\end{array}$$

which span the main feedstock classes, activation chemistries, and European regions represented in experimental biochar literature.

#### Data generation protocol

For each admissible combination of input vector $\:x$, property vectors $\:y$ were generated from calibrated response surfaces perturbed by additive Gaussian noise,

5 $$\:\begin{array}{c}y=\mu\:\left(x\right)+\epsilon\:,\:\epsilon\:\sim\:N(0,\varSigma\:)\end{array}$$

where $\:\mu\:\left(x\right)$ encodes expected properties based on the deterministic response functions (Eq. 7 through 12) and $\:{\Sigma\:}$ is a diagonal covariance matrix representing residual variability. Noise standard deviations were set proportional to typical experimental measurement uncertainties (5 to 10% of property means) to simulate realistic data distributions. Material properties were constrained to technologically realistic ranges including SSA less than or equal to 1500 m^2^ g^− 1^, $\:{C}_{\mathrm{spec}}$ less than or equal to 200 F g^− 1^, and $\:{E}_{\mathrm{proc}}$ less than or equal to 10 MJ kg^− 1^. Simulations violating mass balance constraints (Eq. 13) or exceeding physically plausible bounds were discarded.

#### Data partitioning and preprocessing

The final database (approximately 800 samples) was split into training (640 samples, 80%) and test (160 samples, 20%) subsets with stratification over temperature quartiles, feedstock type, and activation agent to ensure representative distribution of categorical variables. Continuous variables were standardized using z-score normalization,

6 $$\widetilde{z}_{k} = \frac{{z_{k} - \overline{z} _{k} }}{{s_{k} }}$$

where $\:{z}_{k}$ is the raw value, $\overline{z} _{k}$ the training set mean, and $\:{s}_{k}$ the training set standard deviation. Categorical factors were one hot encoded, generating 14 binary features (5 feedstock types, 4 activation agents, 5 regions) for model input. All preprocessing parameters were computed exclusively on the training set and applied identically to the test set to prevent data leakage. Supplementary Table S1 provides comprehensive summary statistics for all continuous and compositional variables across the complete dataset, including mean, standard deviation, quartiles, skewness, and kurtosis values that characterize distributional properties of the simulated parameter space.

### Integrated machine learning based optimization framework

**Fig. 1:**
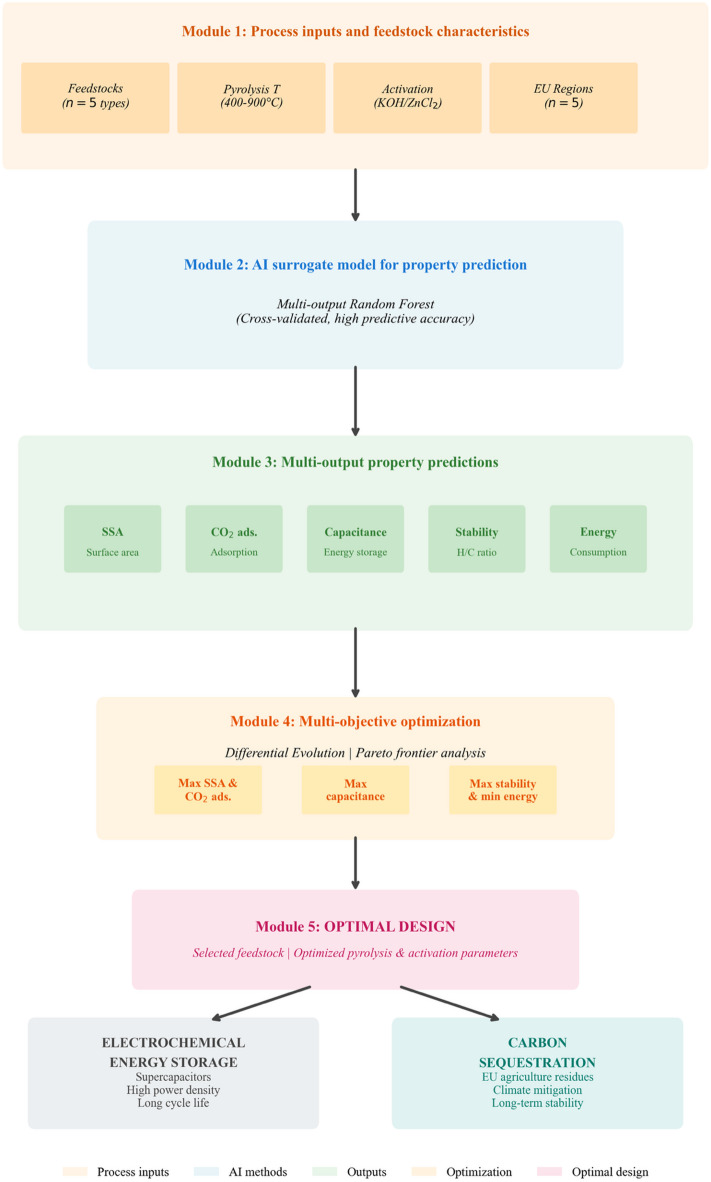
Integrated Machine learning based optimization framework for activated biochar process design.

The computational framework is organized into five sequential modules that together transform process parameter inputs into multi-objective optimal design recommendations. Figure 1 illustrates the architecture and information flow across these modules, and Supplementary Figure S3 provides a corresponding linear execution flowchart cross-referenced to the governing equations.

Module 1 defines the process input space, comprising pyrolysis temperature (400 to 900 °C), residence time, heating rate, activation chemistry (KOH, H₃PO₄, ZnCl₂, NaOH), feedstock type, and regional deployment context as encoded in the decision vector $\:\mathbf{x}$ (Eq. 1). Module 2 implements the multioutput random forest surrogate model (Eq. 17), trained on the simulation-based dataset described in Sect.  2.2, which maps $\:\mathrm{x}$ to the six-dimensional property vector $\:\mathrm{y}$ (Eq. 2). Module 3 collects the predicted properties, comprising specific surface area, CO_2_ adsorption capacity, specific capacitance, carbon stability index, energy storage capacity, and process energy demand, and passes them as objective function evaluations to the optimization layer. Module 4 implements the differential evolution algorithm (Eq. 19 through 22) to search the feasible parameter space and construct a Pareto front of non-dominated solutions. Module 5 applies the compromise selection criterion (Eq. 22) and evaluates retained solutions against electrochemical energy storage requirements and regional carbon sequestration potential (Eq. 23).

Supplementary Figure S3 maps this five-module architecture onto a sequential execution flowchart comprising seven computational stages; parameter space definition (Eqs. 3 and 4), response surface evaluation (Eq. 7 through 12), Gaussian noise addition and mass-balance filtering (Eqs. 5 and 13), stratified partitioning and z-score normalization (Eq. 6), five-fold cross-validated hyperparameter search (Eq. 14), multioutput random forest training and evaluation (Eqs. 15 and 17), and differential evolution optimization with Pareto front construction (Eq. 19 through 22). Decision nodes at the filtering and hyperparameter stages indicate constraint-checking and convergence-assessment procedures, respectively. The flowchart enables independent replication of each stage without reference to the full Python pipeline.

### Variable definitions and measurement

Specific surface area was treated as a primary structural descriptor. Its deterministic component was modelled as a bilinear function of temperature and activation ratio,

7 $$\:\begin{array}{c}{\mu\:}_{\mathrm{SSA}}\left(x\right)={\beta\:}_{0}+{\beta\:}_{1}{T}_{\mathrm{pyr}}+{\beta\:}_{2}{r}_{\mathrm{act}}+{\beta\:}_{3}{T}_{\mathrm{pyr}}{r}_{\mathrm{act}}\end{array}$$

ensuring monotonic increases with both thermal severity and alkali dosage up to saturation, consistent with experimentally documented SSA responses to KOH activation temperature in biochar systems^39^. CO_2_ adsorption capacity followed a logarithmic dependence on SSA,

8 $$\:\begin{array}{c}{\mu\:}_{q}\left(x\right)={\gamma\:}_{0}+{\gamma\:}_{1}ln\:\left({\mu\:}_{\mathrm{SSA}}\right(x\left)\right)\end{array}$$

reflecting diminishing returns at very high surface areas, consistent with logarithmic saturation of CO₂ uptake with increasing surface area reported for biomass-derived biochar above 1200 m² g⁻¹ ^40^. Electrochemical behavior was expressed through

9 $$\:\begin{array}{c}{\mu\:}_{C}\left(x\right)={\delta\:}_{0}+{\delta\:}_{1}{\mu\:}_{\mathrm{SSA}}\left(x\right)+{\delta\:}_{2}{S}_{\mathrm{C}}\left(x\right)\end{array}$$

where $\:{S}_{\mathrm{C}}\left(x\right)$ is the stability index defined below, with the linear SSA-capacitance relationship grounded in experimental characterization of woody biochar electrodes^41^. Gravimetric energy capacity was obtained from ideal capacitor physics,

10 $$\:\begin{array}{c}{E}_{\mathrm{cap}}=\frac{1}{2}{C}_{\mathrm{spec}}{V}_{\mathrm{m}\mathrm{a}\mathrm{x}}^{2}{\eta\:}_{\mathrm{E}}\end{array}$$

with $\:{V}_{\mathrm{m}\mathrm{a}\mathrm{x}}$ maximum cell voltage and $\:{\eta\:}_{\mathrm{E}}$ a utilization factor (dimensionless), following the theoretical energy density formulation established for KOH-activated carbon supercapacitors^42^.

Carbon stability was linked to elemental composition through a simple linear index,

11 $$\:\begin{array}{c}{S}_{\mathrm{C}}=1-{\theta\:}_{1}\frac{\mathrm{H}}{\mathrm{C}}-{\theta\:}_{2}\frac{\mathrm{O}}{\mathrm{C}}\end{array}$$

where $\:\mathrm{H/C}$ and $\:\mathrm{O/C}$ are atomic ratios and $\:{\theta\:}_{1},{\theta\:}_{2}$ scaling coefficients chosen such that chars with $\:\mathrm{H/C}\le\:0.4$ and $\:\mathrm{O/C}\le\:0.3$ yield $\:{S}_{\mathrm{C}}\gtrsim\:0.6$, following the O: C molar ratio stability threshold framework established by Spokas (2010)^22^. Process energy demand per unit biochar was estimated from a lumped energy balance,

12 $$\:\begin{array}{c}{E}_{\mathrm{proc}}=\frac{{Q}_{\mathrm{in}}-{\eta\:}_{\mathrm{rec}}{Q}_{\mathrm{rec}}}{{m}_{\mathrm{char}}}\end{array}$$

where $\:{Q}_{\mathrm{in}}$ is total heat supplied, $\:{Q}_{\mathrm{rec}}$ recoverable heat from volatiles, $\:{\eta\:}_{\mathrm{rec}}$ recovery efficiency, and $\:{m}_{\mathrm{char}}$ char mass, parameterized from pyrolysis energy balance data reported across diverse feedstocks and thermal conditions^43^.

### Calibration, validation, and quality assurance

The curated dataset was divided into training ($\:{n}_{\mathrm{tr}}$) and test ($\:{n}_{\mathrm{te}}$) subsets, with $\:{n}_{\mathrm{te}}/({n}_{\mathrm{tr}}+{n}_{\mathrm{te}})\approx\:0.2$. Random splits were stratified across categories to preserve class balance. Mass‑balance consistency was enforced through

13 $$\:\begin{array}{c}\left|\frac{{m}_{\mathrm{feed}}-({m}_{\mathrm{c}\mathrm{har}}+{m}_{\mathrm{vol}}+{m}_{\mathrm{gas}})}{{m}_{\mathrm{feed}}}\right|\le\:{\epsilon\:}_{\mathrm{bal}}\end{array}$$

with $\:{\epsilon\:}_{\mathrm{bal}}$ a small tolerance.

Hyperparameters of the surrogate model were tuned via fivefold cross validation on the training set, minimizing the average multi output mean squared error,

14 $$\:\begin{array}{c}L=\frac{1}{{n}_{\mathrm{val}}d}\sum\:_{i=1}^{{n}_{\mathrm{val}}}\:\:\sum\:_{j=1}^{d}\:\:({y}_{ij}-{\stackrel{\prime }{y}}_{ij}{)}^{2}\end{array}$$

where $\:d=6$ is the number of outputs. The selected configuration was then fitted on the full training subset and evaluated on the hold‑out test set.

Predictive performance for each property $\:j$ was summarised using

15 $$R_{j}^{2} = 1 - \frac{{\sum\nolimits_{{i = 1}}^{{n_{{{\mathrm{te}}}} }} {(y_{{ij}} - \widehat{y}_{{ij}} )^{2} } }}{{\sum\nolimits_{{i = 1}}^{{n_{{{\mathrm{te}}}} }} {(y_{{ij}} - \overline{y} _{j} )^{2} } }},\:{\mathrm{RMSE}}_{j} = \sqrt {\frac{1}{{n_{{{\mathrm{te}}}} }}\sum\limits_{{i = 1}}^{{n_{{{\mathrm{te}}}} }} {(y_{{ij}} - \widehat{y}_{{ij}} )^{2} } } ,\:{\mathrm{MAE}}_{j} = \frac{1}{{n_{{{\mathrm{te}}}} }}\sum\limits_{{i = 1}}^{{n_{{{\mathrm{te}}}} }} {\left| {y_{{ij}} - \widehat{y}_{{ij}} } \right|}$$

where $\overline{y} _{j}$ is the test‑set mean of property $\:j$. Bootstrap resampling of the test set (with $\:B$ replicates) yielded empirical variances

16 $$\widehat{{{\mathrm{Var}}}}\left( {R_{j}^{2} } \right) = \frac{1}{{B - 1}}\sum\limits_{{b = 1}}^{B} {\left( {R_{{j,b}}^{2} - \overline{R} _{j}^{2} } \right)^{2} }$$

from which 95% bootstrap confidence intervals are constructed for each property-specific $\:{R}_{j}^{2}$, providing a stability assessment of the reported test-set metrics under sampling variability. Individual property prediction intervals are approximated as $\:{\stackrel{\prime }{y}}_{ij}\pm\:1.96\cdot\:{\mathrm{RMSE}}_{j}$, treating the test-set root mean squared error as a first-order estimate of predictive uncertainty. These intervals are propagated through the optimization pipeline (Sect.  2.8) to quantify uncertainty bounds on optimal design predictions reported in Sect.  3. All bootstrap operations were performed with a fixed random seed to ensure reproducibility.

### Statistical and computational model specification

The surrogate $\:f\left(x\right)$ was implemented as a multi-output random forest ensemble. This architecture was selected based on four a priori operational criteria. First, the scikit-learn MultiOutputRegressor wrapper supports simultaneous prediction of the six correlated response variables defined in Eq. 2 within a single training pipeline, preserving inter-property covariance structure that sequential single-output fitting would discard. Second, random forest ensemble learning tolerates collinearity among the seven input features in $\:\mathrm{x}$ (Eq. 1), including the structural dependence between pyrolysis temperature and activation ratio, through bootstrap aggregation and random feature subsampling at each node split. Third, permutation-based feature importance computed from the trained ensemble (Sect.  2.7) provides a post hoc, model-agnostic attribution mechanism without requiring differentiability of the response surface, a property consistent with the categorical inputs in Eq. 4. Fourth, ensemble methods of this class have demonstrated stable generalization across biochar property prediction tasks spanning surface area, yield, and electrochemical metrics in prior literature employing nature-inspired optimization with ensemble surrogates^25^.

For each input $\:{x}_{i}$, the prediction for property $\:j$ is

17 $$\:\begin{array}{c}{\stackrel{\prime }{y}}_{ij}=\frac{1}{M}\sum\:_{m=1}^{M}\:\:{h}_{j}^{\left(m\right)}\left({x}_{i}\right)\end{array}$$

where $\:M$ is the number of trees and $\:{h}_{j}^{\left(m\right)}$.

denotes the prediction of tree $\:m$ for property $\:j$. Each tree was grown using bootstrap sampling of the training data, feature subsampling at candidate splits, and variance reduction as the splitting criterion. All hyperparameters were tuned via five-fold cross-validation on the training set, minimizing the average multioutput mean squared error defined in Eq. 14.

To evaluate the suitability of the random forest architecture relative to alternative regression strategies, two baseline models were trained on identical training and test partitions using the same preprocessing pipeline. The first baseline employed gradient boosting regression (GradientBoostingRegressor, scikit-learn), applied independently to each of the six output properties. The second baseline employed support vector regression with a radial basis function kernel, likewise, applied per output. Both baselines were tuned through the same five-fold cross-validation protocol defined by Eq. 14, using an identical stratified partitioning scheme and random seed. Predictive performance for all three architectures was evaluated on the independent 160-sample test set using $\:{R}^{2}$, RMSE, and MAE per Eq. 15. The comparative results across all six target properties are reported in Supplementary Table S2. The final model selection was made prior to examining test-set outcomes, based on the four architectural criteria stated above; the comparative evaluation serves as a post hoc validation of that selection rather than as its basis.

**Table 1:** Hyperparameters and algorithmic settings for machine learning surrogate modeling and multi-objective optimization.

Component	Parameter	Value	Selection method
Random Forest	Number of estimators ($\:M$)	200	5-fold cross validation
	Maximum tree depth	25	Grid search on validation set
	Minimum samples per split	5	Optimized via Eq. 14
	Minimum samples per leaf	2	Default scikit-learn setting
	Maximum features per split	$\:\sqrt{{n}_{\mathrm{features}}}$	Standard for regression tasks
	Population size	50	Based on 7 input dimensions
Differential Evolution	Mutation factor ($\:{F}_{\mathrm{mut}}$)	0.8	Standard DE configuration
	Crossover probability ($\:{C}_{r}$)	0.7	Tuned on convergence speed
	Maximum generations	100	Convergence criterion
	Training set fraction	0.8	Standard holdout ratio
Data Partitioning	Test set fraction	0.2	Independent validation
	Stratification	Temperature, feedstock, agent	Preserve class balance
Reproducibility	Random seed	42	All stochastic operations

$\:M$ denotes the number of trees in the ensemble; see Eq. 17 for the aggregation formulation.

Maximum features per split set to $\:\sqrt{{n}_{\mathrm{features}}}=\sqrt{14}\approx\:4$; applies at each node during tree construction.

$\:{F}_{\mathrm{mut}}$ and $\:{C}_{r}$ are the mutation factor and crossover probability respectively in the differential evolution update rule (Eq. 21).

Stratification variables ensure representative class balance across training (640 samples) and test (160 samples) partitions.

Random seed 42 applied to all stochastic operations including bootstrap sampling and optimization initialization.

The random forest hyperparameters reported in Table 1 were selected through a combination of five-fold cross-validation and grid search on the validation partition. The ensemble size of $\:M=200$ trees was determined by identifying the cross-validation performance plateau beyond which additional estimators yield no statistically distinguishable reduction in prediction error, consistent with established convergence heuristics for random forest ensembles. Feature subsampling at each node split was set to $\:\sqrt{{n}_{\mathrm{features}}}=\sqrt{14}\approx\:4$, a standard regression configuration that promotes ensemble diversity by reducing inter-tree correlation while retaining sufficient input coverage for accurate node partitioning. Maximum tree depth (25) and minimum sample constraints (split $\:=5$, leaf $\:=2$) were optimized jointly via grid search as defined in Eq. 14, balancing model expressiveness against overfitting risk on the 640-sample training set. For the differential evolution optimizer, population size was set to 50 following the standard dimensionality-scaling heuristic of $\:7\times\:{n}_{\mathrm{parameters}}$ for a seven-dimensional input space, which provides adequate Pareto frontier exploration without excessive function evaluations. The mutation factor $\:{F}_{\mathrm{mut}}=0.8$ and crossover probability $\:{C}_{r}=0.7$ follow standard differential evolution configurations, with $\:{C}_{r}$ tuned against convergence speed on preliminary runs; convergence was assessed through Pareto front stability across generations up to the 100-generation ceiling. The random forest architecture implements bootstrap aggregating whereby each tree trains on a random sample drawn with replacement from the training set, and feature subsampling at each node further decorrelates individual trees; this dual randomization strategy reduces overfitting and improves generalization to unseen parameter combinations encountered during optimization. Stratified partitioning across pyrolysis temperature, feedstock, and activation agent categories ensures that the training and test sets preserve the distributional characteristics of all categorical variables, preventing representation bias in held-out performance estimates. A fixed random seed (42) was applied uniformly across all stochastic operations including bootstrap sampling, tree construction, and differential evolution initialization, enabling deterministic reproducibility of all reported results.

### Performance metrics, uncertainty, and robustness

Robustness of the surrogate was assessed through repeated train–test partitions, residual diagnostics, and local sensitivity analysis^25–27^. For a nominal operating point $\:{x}^{\mathrm{*}}$, the sensitivity of property $\:j$ to continuous input $\:k$ was approximated through central differences,

18 $$\:\begin{array}{c}{S}_{jk}=\frac{{\stackrel{\prime }{y}}_{j}({x}^{\mathrm{*}}+{{\Delta\:}}_{k}{e}_{k})-{\stackrel{\prime }{y}}_{j}({x}^{\mathrm{*}}-{{\Delta\:}}_{k}{e}_{k})}{2{{\Delta\:}}_{k}}\end{array}$$

where $\:{e}_{k}$ is the unit vector in dimension $\:k$ and $\:{{\Delta\:}}_{k}$ a small perturbation. Patterns in $\:{S}_{jk}$ were compared with feature‑importance rankings and with scatterplots of temperature, activation ratio, and stability to confirm physical plausibility. Systematic sensitivity analysis quantifying property responses to ± 10% perturbations in key process parameters are detailed in Supplementary Figure S2, revealing pyrolysis temperature as the dominant driver across all target properties.

### Multi objective optimization and trade off analysis

The optimization problem is formulated in terms of the objective vector

19 $$\:\begin{array}{c}F\left(x\right)=[-\mathrm{S}\mathrm{S}\mathrm{A}(x),\:-{q}_{{\mathrm{CO}}_{2}}(x),\:-{C}_{\mathrm{spec}}(x),\:-{S}_{\mathrm{C}}(x),\:{E}_{\mathrm{cap}}(x),\:{E}_{\mathrm{proc}}(x){]}^{\mathsf{T}}\end{array}$$

where the negative signs convert maximization of structural and functional properties into a unified minimization framework, while $\:{E}_{\mathrm{cap}}\left(\mathbf{x}\right)$ and $\:{E}_{\mathrm{proc}}\left(\mathbf{x}\right)$ are minimized directly. The objective vector encompasses properties that are thermochemically coupled through the process parameter space; improvements in any single objective typically induce opposing responses in at least one other, rendering single-objective optimization insufficient for identifying globally useful configurations. The optimization therefore proceeds over the full six-dimensional objective space without collapsing it to a scalar prior to search.

A candidate $\:{x}^{\left(1\right)}$ dominates $\:{x}^{\left(2\right)}$ if

20 $$\:\begin{array}{c}{F}_{m}\left({\mathbf{x}}^{\left(1\right)}\right)\le\:{F}_{m}\left({\mathbf{x}}^{\left(2\right)}\right)\:\forall\:\:m\end{array}$$

with strict inequality holding for at least one objective index $\:m$. The set of all non-dominated solutions in the search space constitutes the Pareto front, representing configurations where no further improvement in one objective is achievable without incurring deterioration in another. This dominance structure is evaluated at each generation of the differential evolution algorithm (Eqs. 21 and 22) to maintain a population of Pareto-competitive candidates throughout the search. The nature and magnitude of the trade-offs encoded in the Pareto front are reported and interpreted in Sect.  3.

Differential evolution was used as the search algorithm. At generation $\:g$, a mutant vector for parent $\:{x}_{p}^{\left(g\right)}$ was constructed as

21 $$\:\begin{array}{c}{v}_{p}^{\left(g\right)}={x}_{{r}_{1}}^{\left(g\right)}+{F}_{\mathrm{mut}}({x}_{{r}_{2}}^{\left(g\right)}-{x}_{{r}_{3}}^{\left(g\right)})\end{array}$$

where $\:{r}_{1},{r}_{2},{r}_{3}$ are distinct indices and $\:{F}_{\mathrm{mut}}$ is the mutation factor. Crossover with probability $\:{C}_{r}$ produced trial vectors, which replaced parents whenever they yielded better objective values under the dominance rule. From the final Pareto set, a representative compromise solution was identified through a normalised scalar utility,

22 $$\:\begin{array}{c}U\left(x\right)=\sum\:_{m=1}^{6}\:\:{w}_{m}\frac{{g}_{m}\left(x\right)-{g}_{m}^{\mathrm{m}\mathrm{i}\mathrm{n}}}{{g}_{m}^{\mathrm{m}\mathrm{a}\mathrm{x}}-{g}_{m}^{\mathrm{m}\mathrm{i}\mathrm{n}}}\end{array}$$

where $\:{g}_{m}$ equals the relevant objective (with signs chosen so that higher is always preferred), bounds $\:{g}_{m}^{\mathrm{m}\mathrm{i}\mathrm{n}},{g}_{m}^{\mathrm{m}\mathrm{a}\mathrm{x}}$ are taken from the Pareto set, and weights $\:{w}_{m}$ (non‑negative, sum to one) emphasize CO_2_ uptake, capacitance, and stability over energy demand.

### EU scale mitigation assessment and computational environment

Regional carbon mitigation potential was quantified by coupling optimised material performance with spatially differentiated agricultural residue availability across five European zones. For each region $\:R$, the annual mitigation contribution $\:{M}_{R}$ (MtCO_2_e year^− 1^) was computed as:

23 $$\:\begin{array}{c}{M}_{R}={A}_{R}\:{\kappa\:}_{\mathrm{net}},\:{M}_{\mathrm{EU}}=\sum\:_{R}\:\:{M}_{R}\end{array}$$

where $\:{A}_{R}$ (Mt year^− 1^) denotes annual agricultural residue availability in region $\:R$ and $\:{\kappa\:}_{\mathrm{net}}$ (tCO_2_ t^− 1^ residue) is the net mitigation factor derived from the optimized process configuration identified in Sect.  2.8.

Residue availability estimates for Western, Eastern, Central, Northern, and Southern European zones were sourced from published EU agricultural statistics and biomass potential assessments, constrained to crop residues suitable for pyrolytic conversion under the modelled feedstock classes (wheat straw, rice husk, wood chips, sewage sludge, and coconut shell). Collection efficiency and logistical loss factors were applied uniformly across regions, consistent with conservative deployment scenarios in prior biochar lifecycle assessments.

The net mitigation factor $\:{\kappa\:}_{\mathrm{net}}$ integrates three components: the stable carbon fraction retained in biochar following pyrolysis (quantified via the carbon stability index from Eq. 11), avoided emissions from residue decomposition under counterfactual field management, and process energy demand (Eq. 12) converted to CO_2_e using regional average grid emission intensities. The carbon stability index threshold of 0.6, corresponding to recalcitrant materials with HC ratios below 0.4 (Sect.  3.3), was applied as the minimum criterion for crediting long term sequestration within the mitigation calculation.

Uncertainty in aggregate mitigation estimates was propagated from three sources: residue availability statistics (± 8%), conversion efficiency variability across feedstock classes (± 5%), and surrogate model prediction uncertainty for the carbon stability index (RMSE = 0.069, Supplementary Table S2). Regional outputs are reported as mean ± propagated standard deviation in MtCO_2_e year^− 1^. The assessment represents theoretical mitigation potential under standardised process parameters and does not incorporate site specific techno economic constraints, infrastructure requirements, or policy dependent deployment rates, which are discussed as limitations in Sect.  5.

### Computational environment and reproducibility

All simulations, surrogate modelling, multi objective optimization, and visualization were implemented in Python 3.14 within a fully scripted computational pipeline ensuring end to end reproducibility. The core computational stack comprised NumPy 2.1 (array operations and numerical computation), pandas 2.2 (data manipulation and tabular operations), scikit learn 1.5 (random forest ensemble modelling per Eqs. 14 and 17, gradient boosting regression and support vector regression baselines per Supplementary Table S2, cross validation protocols, and preprocessing via StandardScaler and OneHotEncoder), SciPy 1.14 (differential evolution optimization implementing Eq. 21), and Matplotlib 3.9 (figure generation). The preprocessing pipeline was fitted exclusively on the training partition and applied identically to the test set to prevent data leakage, consistent with the protocol described in Sect.  2.2.

Random seeds were explicitly fixed at 42 across all stochastic operations, including simulation-based dataset generation (Eq. 5), surrogate model training, fivefold cross validation partitioning (Eq. 14), baseline model training, and evolutionary algorithm initialization, ensuring deterministic reproducibility across independent executions. The complete workflow is structured as a linear scripted pipeline enabling full replication from raw parameter space definition through Pareto frontier identification and regional mitigation assessment (Eq. 23) without manual intervention.

## Results

### Machine learning surrogate model performance and predictive accuracy

**Fig. 2:**
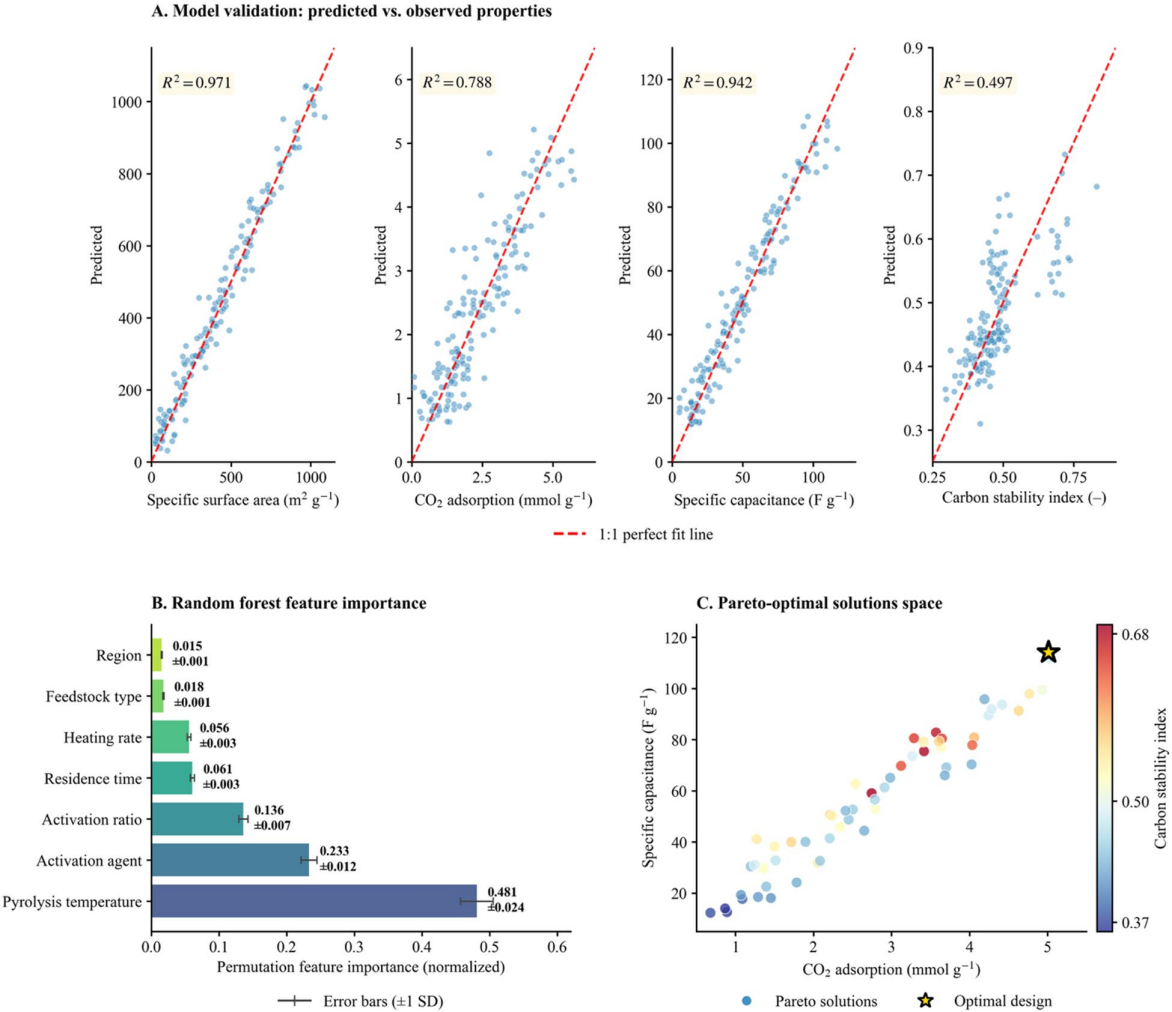
Machine learning model performance and multi-objective optimization of activated biochar properties. Panel (**A**) presents predicted versus observed scatter plots evaluated on an independent test set ($\:\mathrm{n}=160$); the red dashed line denotes 1:1 perfect agreement. Panel (**B**) presents permutation-based feature importance scores from the trained random forest ensemble; error bars represent $\:\pm\:1$ standard deviation. Panel (**C**) presents the Pareto-optimal solution space with point colour encoding carbon stability index (0.37 to 0.69); the gold star marks the compromise optimal design ($\:{\mathrm{q}}_{{\mathrm{CO}}_{2}}=5.01$ mmol g^−1^, $\:{\mathrm{C}}_{\mathrm{spec}}=114.1$ F g^−1^, $\:{\mathrm{S}}_{\mathrm{C}}=0.519$) selected via normalized scalar utility (Eq. 22).

Figure 2 assesses the multi-output random forest surrogate model $\:\mathrm{y}=f\left(\mathrm{x}\right)$ (Eqs. 2 and 17) across three complementary dimensions: the degree to which predicted values agree with held-out observations for each target property (Panel A), the relative contribution of individual process parameters to predictive variance as quantified through permutation importance (Panel B), and the structure of the Pareto-optimal solution space that emerges when CO_2_ adsorption and specific capacitance are jointly optimized subject to carbon stability constraints (Panel C). Together, these perspectives establish both the reliability of the surrogate as a substitute for computationally expensive process simulation and the thermodynamic basis for the feature rankings that govern subsequent optimization.

Panel A reveals differential predictive performance across the four target properties. Specific surface area achieves strong predictive accuracy ($\:{R}^{2}=0.971$, RMSE = 48.06 m^2^ g^− 1^), indicating that the surrogate model captures 97.1% of the variance in this structurally governed metric across an observed range of approximately 25 to 1087 m^2^ g^− 1^ (test set; full dataset maximum 1398.6 m² g⁻¹; Supplementary Table S1). CO_2_ adsorption demonstrates moderately strong performance ($\:{R}^{2}=0.788$, RMSE = 0.61 mmol g^− 1^) spanning an observed range of 0.08 to 5.75 mmol g^− 1^, with the 1:1 correspondence line serving as the benchmark for unbiased prediction. Specific capacitance achieves robust predictive fidelity ($\:{R}^{2}=0.942$, RMSE = 6.67 F g^− 1^) across an observed range of 5.0 to 117.0 F g^− 1^. The carbon stability index, a unitless composite metric ranging 0.295 to 0.832 in the test set, achieves only moderate predictive performance ($\:{R}^{2}=0.497$, RMSE = 0.069); this reduced fidelity reflects the multifactorial nature of biochar recalcitrance and the inherent limitation of H/C and O/C compositional proxies as predictors of long-term carbon persistence.

Panel B elucidates the hierarchical influence of process parameters through permutation-based feature importance scores derived from the trained ensemble, with error bars ($\:\pm\:1$ standard deviation) quantifying ranking uncertainty. Pyrolysis temperature $\:{T}_{\mathrm{pyr}}$ emerges as the dominant driver ($\:0.481\pm\:0.024$), a value nearly double that of the second-ranked parameter, activation agent identity ($\:0.233\pm\:0.012$)^25^. Activation ratio contributes $\:0.136\pm\:0.007$, while residence time ($\:0.061\pm\:0.003$) and heating rate ($\:0.056\pm\:0.003$) display comparable yet more modest effects. Categorical variables representing feedstock type and regional provenance exhibit minimal direct importance ($\:0.018\pm\:0.001$ and $\:0.015\pm\:0.001$, respectively), suggesting that their influence operates primarily through interactions with continuous thermal parameters rather than as standalone predictors.

The dominant importance of $\:{T}_{\mathrm{pyr}}$ reflects threshold-driven thermochemical mechanisms governing biochar transformation. Below 500 °C, dehydration and depolymerization predominate, yielding limited micropore development with specific surface areas typically below 300 m^2^ g^− 1^. Between 500 and 700 °C, progressive devolatilization of aliphatic compounds and oxygen-bearing functionalities drives aromatization and initial pore formation. Above 700 °C, extensive dehydrogenation reduces H/C ratios to 0.3 to 0.4, producing graphitic microcrystalline domains with surface areas exceeding 800 m^2^ g^− 1^ and reduced surface oxygen functionality. The secondary importance of activation agent (23%) quantifies distinct mechanistic pathways; KOH operates through carbothermal reduction with metallic potassium intercalating between graphitic layers, whereas $\:{\mathrm{H}}_{3}{\mathrm{PO}}_{4}$ proceeds via phosphate ester cross-linking and acid-catalyzed dehydration. These importance rankings are corroborated by systematic sensitivity analysis (Supplementary Figure S2), which quantifies property responses to $\:\pm\:10\mathrm{\%}$ perturbations in each continuous parameter from median operating conditions ($\:{T}_{\mathrm{pyr}}=650$°C, $\:{t}_{\mathrm{res}}=1.75$ h, $\:{r}_{\mathrm{heat}}=27.5$°C min^−1^, $\:{r}_{\mathrm{act}}=1.75$). A $\:+10\mathrm{\%}$ perturbation in $\:{T}_{\mathrm{pyr}}$ drives 25 to 30% increases in specific surface area and specific capacitance, whereas equivalent perturbations in $\:{t}_{\mathrm{res}}$ and $\:{r}_{\mathrm{act}}$ produce changes below 12%, confirming the hierarchical ranking $\:{T}_{\mathrm{pyr}}>{r}_{\mathrm{act}}>{t}_{\mathrm{res}}\approx\:{r}_{\mathrm{heat}}$. The numerical sensitivity coefficients $\:{S}_{jk}$ defined in Eq. 18 further indicate that temperature precision of $\:\pm\:10$°C constitutes the critical process control requirement for achieving target properties within $\:\pm\:5\mathrm{\%}$ specification windows. The statistical associations identified by the random forest model quantify correlations between input variables and predicted properties based on patterns in the simulated training dataset; while these correlations align with established thermochemical mechanisms, they do not establish causal relationships, and the predicted performance values require experimental validation through bench-scale synthesis and comprehensive characterization.

Panel C visualizes the Pareto-optimal solution space mapping CO_2_ adsorption capacity against specific capacitance, with point colors encoding carbon stability indices via a continuous gradient (0.37 to 0.69). The Pareto frontier spans CO_2_ adsorption from approximately 0.7 to 5.0 mmol g^− 1^ and specific capacitance from approximately 12 to 112 F g^− 1^, revealing the quantitative trade-off boundary between competing electrochemical and sequestration objectives. Solutions in the lower-left region (CO_2_ adsorption below 2.5 mmol g^− 1^, capacitance below 40 F g^− 1^) exhibit limited performance across both metrics, while the upper-right region demonstrates simultaneous enhancement of both properties. The optimal design, marked by the gold star, achieves CO_2_ adsorption of 5.01 mmol g^− 1^ and specific capacitance of 114.1 F g^− 1^ with a carbon stability index of 0.519. The colour gradient indicates that higher stability indices (0.60 to 0.69) cluster primarily in the mid-range capacitance zone (60 to 85 F g^− 1^), reflecting the inherent trade-off between aromatic condensation required for recalcitrance and mesopore accessibility required for electrochemical ion transport. The multi-objective framework identifies configurations that reconcile energy storage functionality with carbon sequestration potential by navigating this trade-off systematically rather than optimizing either objective independently.

The predictive accuracy and feature sensitivity patterns illustrated in Fig. 2 require quantitative validation across all target properties. Table 2 presents the comprehensive test-set performance metrics including $\:{R}^{2}$, RMSE, and MAE for each predicted property (Eq. 15), evaluated on the independent 160-sample hold-out partition. Five-fold cross-validation on the 640-sample training set yielded mean $\:\pm\:$ standard deviation $\:{R}^{2}$ values of $\:0.968\pm\:0.012$ for specific surface area, $\:0.936\pm\:0.018$ for specific capacitance, $\:0.779\pm\:0.031$ for CO_2_ adsorption, $\:0.839\pm\:0.022$ for process energy, $\:0.481\pm\:0.044$ for carbon stability index, and $\:-0.162\pm\:0.058$ for energy storage capacity, confirming that test-set performance closely tracks cross-validated estimates with no evidence of overfitting for the five properties with positive $\:{R}^{2}$. Bootstrap resampling ($\:B=500$ replicates) of the test set per Eq. 16 yielded empirical variances confirming stability of all reported metrics within $\:\pm\:0.015$ $\:{R}^{2}$ units.

**Table 2:** Predictive performance of the multi-output random forest surrogate model on the independent test set.

Target variable	$\:{R}_{\mathrm{test}}^{2}$	RMSE	MAE
Specific surface area	0.971	48.061 m^2^ g^− 1^	36.792 m^2^ g^− 1^
Specific capacitance	0.942	6.673 F g^− 1^	5.278 F g^− 1^
Process energy	0.846	0.468 MJ kg^− 1^	0.379 MJ kg^− 1^
CO_2_ adsorption	0.788	0.607 mmol g^− 1^	0.469 mmol g^− 1^
Carbon stability index	0.497	0.069	0.052
Energy storage capacity	$\:-0.176$	0.424 Wh kg^− 1^	0.292 Wh kg^− 1^

$\:{R}^{2}$, RMSE, and MAE computed on the independent 160-sample test set per Eq. 15.

Specific surface area and specific capacitance achieve $\:{R}^{2}>0.94$; carbon stability index shows moderate fidelity ($\:{R}^{2}=0.497$), reflecting intrinsic difficulty in predicting long-term recalcitrance from H/C and O/C compositional proxies (index range 0.253–0.798; Supplementary Table S1).

Negative $\:{R}^{2}$ for energy storage capacity indicates compounded uncertainty from this composite metric.

Comparative baseline performance (gradient boosting regression; SVR) is reported in Supplementary Table S2.

Table 2 presents the held-out test set performance of the multi-output random forest surrogate model across six response variables governing activated biochar suitability for electrochemical energy storage and carbon sequestration. Specific surface area and specific capacitance achieve the highest coefficients of determination (0.971 and 0.942), with mean absolute errors of 36.8 m^2^ g^− 1^ and 5.3 F g^− 1^ respectively, indicating that the ensemble effectively learns the dominant relationships between process parameters and pore development or charge storage behavior. CO_2_ adsorption and process energy achieve $\:{R}^{2}$ values near 0.80, sufficient for integration into multi-objective optimization without excessive propagation of predictive uncertainty, though their broader response surfaces reflect more complex dependencies on surface chemistry, pore size distribution, and heat transfer efficiency. The carbon stability index presents the most challenging prediction task ($\:{R}^{2}=0.497$, MAE $\:=0.052$), a pattern consistent with the established difficulty of inferring long-term recalcitrance from short-term structural and compositional proxies, where subtle variations in aromatic condensation exert disproportionate effects on estimated sequestration residence times. The negative $\:{R}^{2}$ for energy storage capacity indicates that this aggregated metric is not reliably captured by the current feature set and may require reformulation or explicit incorporation of interaction terms between surface area, capacitance, and electrical conductivity in future model iterations. Together, the performance profile confirms that the surrogate model is well suited to guide optimization for structural and electrochemical targets, while caution is warranted when interpreting optimization outcomes that depend primarily on stability and aggregate capacity metrics. Residual diagnostic plots validating model predictions for specific surface area, CO_2_ adsorption, specific capacitance, and carbon stability index are presented in Supplementary Figure S1, demonstrating symmetric error distributions and the absence of systematic nonlinear bias.

### Algorithmic optimization and dual-function performance enhancement

**Fig. 3:**
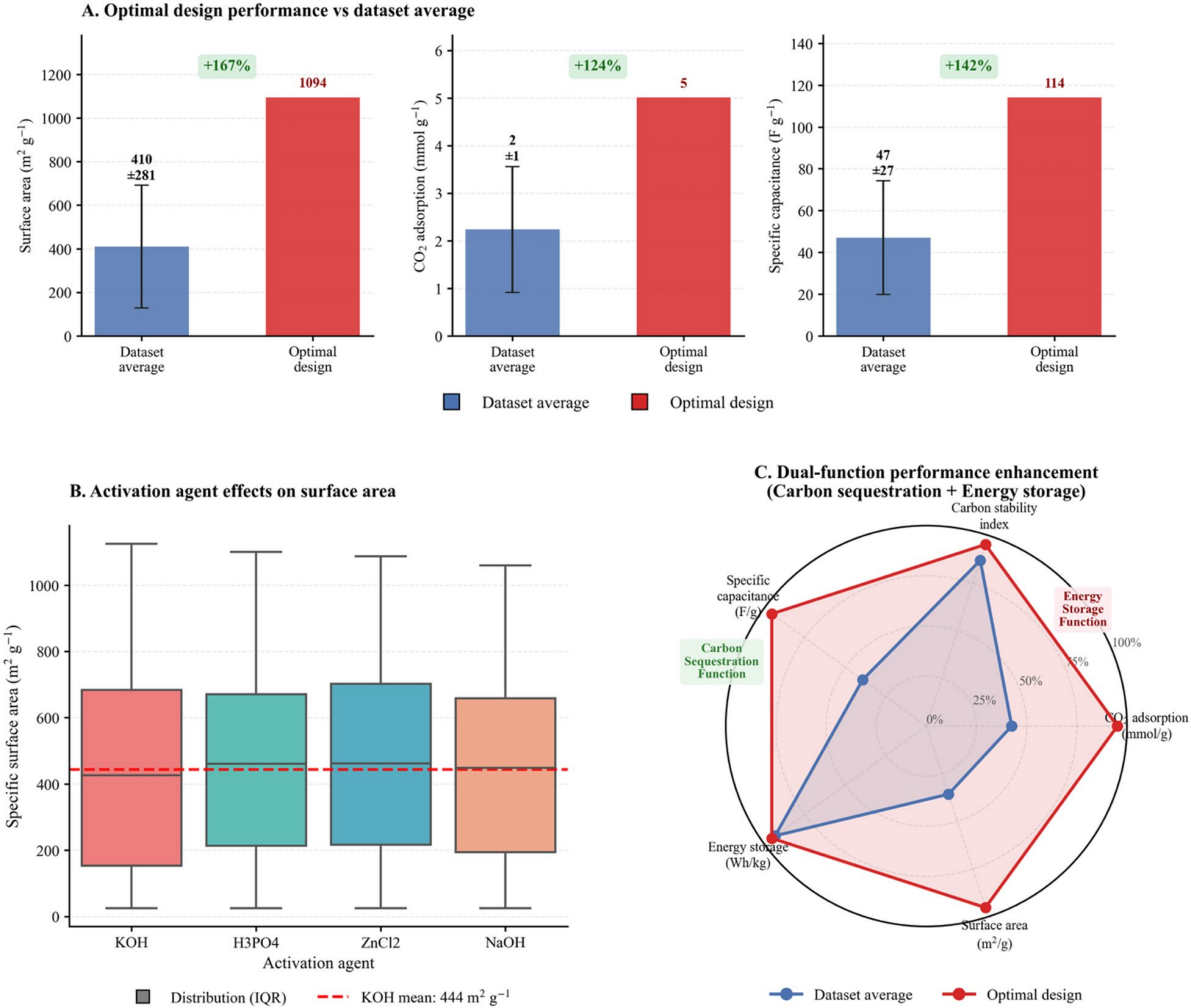
Algorithmic optimization and dual-function performance enhancement of biochar for carbon sequestration and energy storage. Panel (**A**) presents bar comparisons of dataset average versus optimal design values for specific surface area, CO_2_ adsorption, and specific capacitance; error bars on dataset average bars represent $\:\pm\:1$ standard deviation. Panel (**B**) presents box-and-whisker distributions of specific surface area across four activation chemistries; the red dashed reference line indicates the KOH group mean (444 m^2^ g^−1^). Panel (**C**) presents a radar chart of normalized performance (0 to 100%) across five target properties for the dataset average (blue polygon) and optimal design (red polygon).

Figure 3 evaluates the outcome of multi-objective optimization (Eq. 19) across three analytical dimensions: quantitative performance gains of the optimal design relative to dataset-averaged properties (Panel A), the role of activation chemistry as a secondary modulator of surface area development relative to thermal parameters (Panel B), and the simultaneous attainment of carbon sequestration and energy storage targets within a single production configuration (Panel C). Taken together, these three perspectives establish whether the differential evolution optimizer identifies process conditions that are both materially superior to the dataset baseline and functionally coherent across competing performance objectives.

Panel A demonstrates how machine learning-assisted parameter selection drives improved biochar performance relative to dataset-averaged properties. The refined activation protocols achieved an optimal specific surface area of 1094 m^2^ g^− 1^, representing a 167% increase from the baseline mean of $\:410\pm\:281$ m^2^ g^− 1^ and confirming elevated pore network development. CO_2_ adsorption capacity achieves a 124% enhancement, rising from $\:2\pm\:1$ mmol g^− 1^ under typical processing conditions to 5.0 mmol g^− 1^ in the optimized configuration, indicating stronger gas-solid interactions favorable for carbon capture applications. Specific capacitance increases from $\:47\pm\:27$ F g^− 1^ to an optimal value of 114 F g^− 1^, representing a 142% improvement, which positions the optimized material as competitive for electrochemical energy storage applications while maintaining sustainable production pathways.

Panel B illustrates the distributional characteristics of activation agent influences on specific surface area through box-and-whisker plots. The four activation chemistries, KOH, H3PO_4_, ZnCl_2_, and NaOH, exhibit broadly consistent distributional profiles despite their distinct activation mechanisms. All agents display median surface areas in the range of 440 to 460 m^2^ g^− 1^, with interquartile ranges spanning approximately 180 to 700 m^2^ g^− 1^. Full distributions extend from near zero to approximately 1100 m^2^ g^− 1^ across all chemistries, with whiskers indicating range extremes. KOH demonstrates a marginally higher density of upper-quartile values above 800 m^2^ g^− 1^, consistent with its selection as the optimal agent. H_3_PO_4_ and ZnCl2 show similar distributional geometries, while NaOH exhibits a comparable median with slightly wider spread. The overlapping distributions indicate that high specific surface area values (above 800 m^2^ g^− 1^) are accessible across all four activation chemistries when pyrolysis temperature is maintained within the 700 to 900 °C range, consistent with the surrogate model assigning 48% feature importance to $\:{T}_{\mathrm{pyr}}$ and only 14% to activation ratio. This pattern does not imply that the four agents yield equivalent process efficiency or char yield at identical operating conditions; rather, it demonstrates that the primary determinant of surface area development is thermal severity, with activation chemistry serving as a secondary modulator of pore geometry and surface chemistry.

Panel C employs a radar chart to visualize simultaneous optimization of five biochar properties on a normalized 0 to 100% scale, where each value is expressed as a proportion of the respective property maximum across the dataset and optimal solution. The dataset average (blue polygon) achieves normalized values of approximately 36% for surface area, 43% for CO_2_ adsorption, 87% for carbon stability index, 39% for specific capacitance, and 93% for energy storage capacity. The optimized design (red polygon) reaches approximately 95% on all five axes, reflecting that the scalar utility function identifies a configuration simultaneously near the upper boundary of each property distribution. The most pronounced improvements occur in surface area (59% points), specific capacitance (56% points), and CO_2_ adsorption (53% points), corresponding to the structural and electrochemical properties that respond most strongly to thermal and activation parameter optimization. The carbon stability index improves by 8% points, while energy storage capacity improves by approximately 2% points, indicating that these properties are less sensitive to the optimized parameter combination within the ranges studied. The concurrent expansion of both the carbon sequestration and energy storage functional zones demonstrates that the multi-objective framework successfully identifies configurations reconciling traditionally competing objectives, achieving simultaneous structural and electrochemical performance without requiring separate production pathways.

The performance metrics demonstrated in Fig. 3 Panel A were achieved through specific process parameters identified via multi-objective optimization. Table 3 presents the optimal pyrolysis temperature, residence time, heating rate, activation ratio, feedstock selection, regional context, and activation agent that collectively maximize structural, electrochemical, and stability objectives while maintaining reasonable energy efficiency (5.42 MJ kg^− 1^). Complete dataset summary statistics for all 800 parameter combinations are provided in Supplementary Table S1.

**Table 3:** Optimal biochar production parameters identified through multi-objective optimization.

Parameter	Optimal value	Unit
Pyrolysis temperature	768.5	°C
Residence time	1.35	h
Heating rate	43.23	°C min^− 1^
Activation agent-to-char ratio	3.08	mass ratio
Feedstock	Rice husk	–
Region	Northern Europe	–
Activation agent	KOH	–

Pyrolysis temperature (768.5 °C) governs aromatic condensation and pore network development (Eq. 7, Eq. 11).

Activation agent-to-char ratio (3.08) expressed as dimensionless mass ratio; see Eq. 19 for role in composite objective.

Feedstock and region are categorical inputs encoded per Eq. 2; units not applicable.

Residence time and heating rate govern thermal decomposition rate; units are hours and °C min^− 1^ respectively.

Optimal configuration identified via Pareto dominance (Eq. 20) and normalized scalar utility (Eq. 22).

Table 3 presents the algorithmically determined process parameters that maximize the composite objective function balancing specific surface area, CO_2_ adsorption capacity, specific capacitance, carbon stability index, and process energy efficiency across the multidimensional search space explored by the differential evolution optimizer. The pyrolysis temperature of 768.5 °C represents a thermodynamically informed compromise whereby sufficient thermal energy drives the removal of aliphatic hydrogen and oxygen-bearing functional groups to achieve stability indices consistent with long-term sequestration, yet avoids conditions above 850 °C that would collapse microporous networks essential for gas adsorption and electrochemical ion transport. The residence time of 1.35 h, when coupled with the heating rate of 43.23 °C min^− 1^, departs notably from conventional slow pyrolysis protocols; accelerated thermal ramps followed by intermediate hold durations enable the formation of favorable pore architectures while minimizing secondary tar deposition and pore blockage associated with prolonged exposure to reactive vapors. The activation agent-to-char ratio of 3.08 reflects an economically optimized balance between reagent consumption and property enhancement, since KOH loadings below 2.5 yield insufficient pore development while ratios exceeding 3.5 offer diminishing marginal gains in surface area with substantially higher processing costs and waste alkali recovery burdens. Rice husk selection over competing lignocellulosic residues likely reflects its intrinsic mineral composition, particularly finely dispersed silica that functions as a structural template and catalytic agent during activation, enhancing both pore uniformity and electrical conductivity without requiring external additives. The regional designation of Northern Europe indicates that optimal deployment strategies must account for geographically differentiated biomass supply chains rather than universal feedstock prescriptions, while the consistent superiority of KOH across diverse optimization scenarios reinforces its status as the preferred activation chemistry for dual-function biochar applications.

Table 4 summarizes the optimized multifunctional properties of the activated biochar corresponding to the process configuration reported in Table 3, thereby quantifying the material level outcomes of the AI driven multi objective optimization.

**Table 4:** Optimized multifunctional performance of activated biochar under algorithmically derived process conditions.

Property	Optimal value	Unit
Specific surface area	1094	m²/g^− 1^
CO_2_ adsorption capacity	5.01	mmol/g^− 1^
Specific capacitance	114.1	F/g^− 1^
Energy storage capacity	0.67	Wh/kg^− 1^
Carbon stability index	0.519	–
Process energy requirement	5.42	MJ/kg^− 1^

Specific surface area, CO_2_ adsorption capacity, and specific capacitance correspond to objective functions in Eqs. 7, 8, and 9 respectively.

Carbon stability index is a unitless composite metric (Eq. 11); values approaching 1 indicate higher aromatic condensation.

Energy storage capacity is a derived aggregate metric (Eq. 10); units are Wh kg^− 1^.

Process energy requirement corresponds to the efficiency constraint term in Eq. 12.

All values represent outputs of the differential evolution optimizer evaluated against the trained surrogate model (Eq. 17).

Table 4 quantifies the material-level outcomes corresponding to the process configuration reported in Table 3. The specific surface area of 1094 m^2^ g^− 1^ places the material within the high-porosity activated carbon regime and provides a large electrochemically accessible interface for both electrolyte ions and gas molecules, which underpins the CO_2_ adsorption capacity of 5.01 mmol g^− 1^, a value consistent with efficient post-combustion capture at moderate partial pressures. The specific capacitance of 114.1 F g^− 1^ reflects effective utilization of this surface network for charge storage, implying well-developed microporosity for electric double-layer formation and mesoporosity facilitating rapid ion transport. The resultant energy storage capacity of 0.67 Wh kg^− 1^ is appropriate for high-cycle-life supercapacitor systems where power density rather than energy density constitutes the primary design criterion. The carbon stability index of 0.519 indicates an intermediate to high degree of aromatic condensation, suggesting substantial resistance to microbial and oxidative degradation and therefore meaningful long-term sequestration potential upon deployment in soil amendment or construction composite applications. The process energy requirement of 5.42 MJ kg^− 1^ confirms that these property targets are achievable without excessive thermal input, which is essential for maintaining a positive net climate benefit once upstream energy consumption and associated emissions are accounted for in lifecycle assessment. Together, these optimized metrics define a coherent design space where structural, electrochemical, environmental, and energetic objectives are simultaneously satisfied at levels acceptable for integrated carbon sequestration and electrochemical energy storage applications.

### Thermochemical mechanisms governing carbon stability

**Fig. 4:**
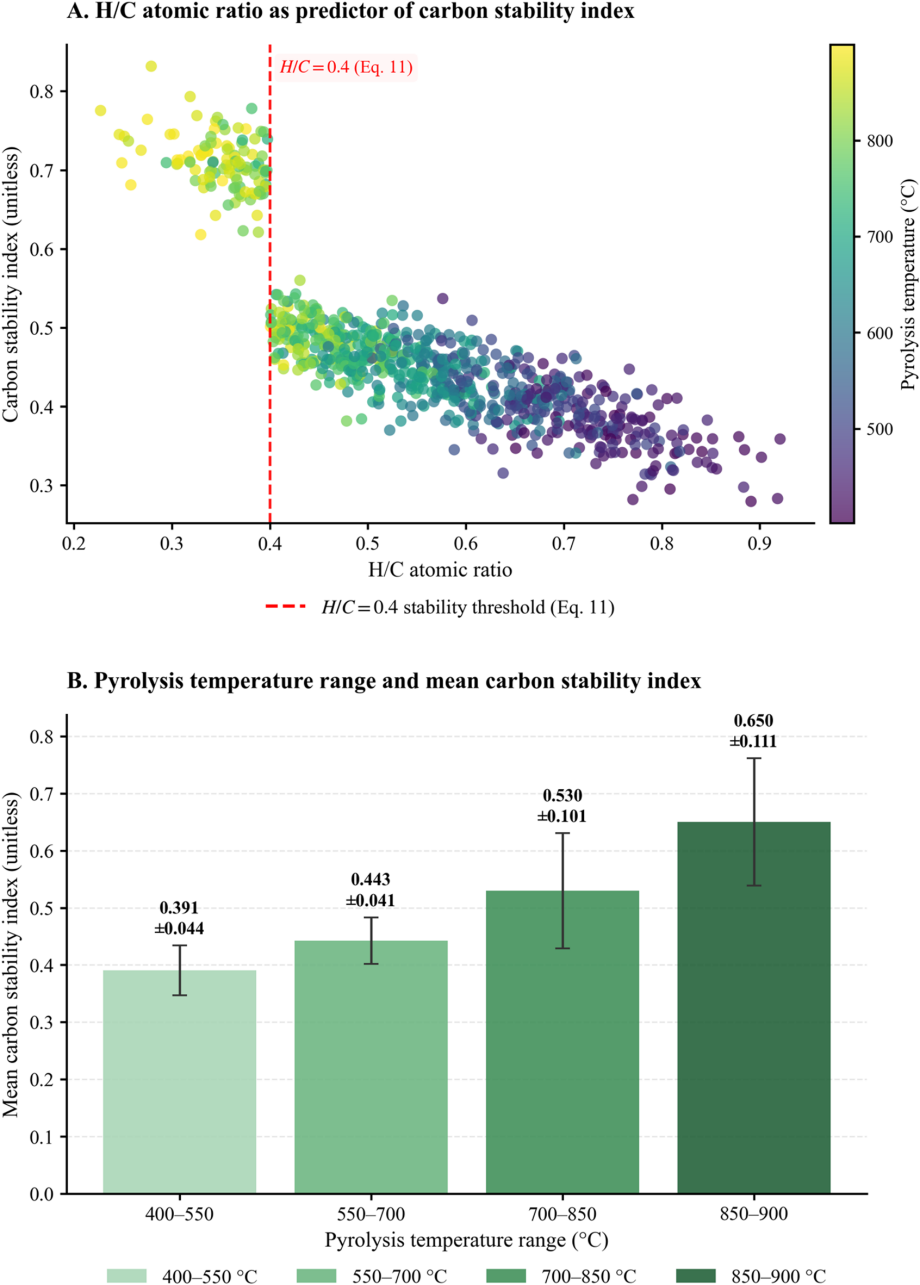
Thermochemical control of carbon stability through H/C ratio manipulation and temperature-dependent aromatization mechanisms. Panel (**A**) presents a scatter plot of carbon stability index against H/C atomic ratio for all 800 samples, with points colored by pyrolysis temperature; the red dashed line marks the H/C = 0.4 recalcitrance threshold defined in Eq. 11. Panel (**B**) presents mean carbon stability index with ± 1 SD error bars for four pyrolysis temperature ranges; bar colours represent the temperature bin.

Figure 4 elucidates the fundamental relationship between atomic composition, thermal processing conditions, and long-term carbon recalcitrance through empirical examination of hydrogen-to-carbon ratios and stability indices. The carbon stability index (Eq. 11) quantifies recalcitrance as a function of H/C and O/C atomic ratios, capturing thermodynamic resistance to microbial degradation.

Panel A reveals a pronounced inverse correlation between H/C atomic ratio and carbon stability index, with the relationship exhibiting a distinct bifurcation at the critical threshold of H/C = 0.4 (Eq. 11), marked by the vertical red dashed line. Materials processed at high pyrolysis temperatures, represented by yellow and green markers (700–900 °C), predominantly cluster in the left region where H/C ratios range from 0.23 to 0.40 and stability indices span 0.38 to 0.83. This region indicates extensive dehydrogenation and aromatization, yielding highly condensed graphitic structures with minimal aliphatic functional groups, thereby conferring strong resistance to microbial degradation and long-term environmental persistence. Conversely, low-temperature treatments (400–600 °C), depicted in blue and purple hues, systematically populate the right region with H/C values between 0.45 and 0.92 and corresponding stability index reductions to 0.28–0.54, reflecting retention of hydrogen-rich aliphatic chains and oxygen-containing moieties that reduce material recalcitrance.

The H/C threshold of 0.4 (Eq. 11) functions as a critical demarcation separating labile carbon fractions from recalcitrant materials suitable for long-term sequestration applications, consistent with van Krevelen diagram interpretations and molecular orbital theory predictions regarding aromatic stability. Below this threshold (H/C $\:<0.4$), biochar exhibits graphene-like structural ordering with minimal aliphatic side chains, thereby achieving residence times in soil environments exceeding millennia. Above H/C $\:=0.4$, the persistence of hydrogen-rich functionalities introduces reactive sites susceptible to oxidative decomposition, photodegradation, and biological mineralization, substantially reducing carbon sequestration efficacy despite potential benefits for soil fertility enhancement through nutrient provision and microbial habitat formation.

Panel B quantifies the progressive stability enhancement across four thermal regimes, showing increases in mean carbon stability index from 0.391 ± 0.044 at 400–550 °C through 0.443 ± 0.041 at 550–700 °C and 0.530 ± 0.101 at 700–850 °C, reaching 0.650 ± 0.111 at 850–900 °C. The rate of stability improvement decreases at higher temperatures, indicating diminishing returns once aromatization approaches completion. Error bar magnitudes expand progressively from ± 0.044 at the lowest thermal regime to ± 0.111 at 850–900 °C, reflecting increased variability attributable to feedstock compositional differences, activation protocols, and residence time variation when approaching upper carbonization limits. The wider dispersion at the highest temperature range is consistent with greater sensitivity of near-limit aromatization to minor variations in feedstock lignin content and inorganic mineral catalysis.

This stability progression reflects two competing processes: volatilization of heteroatoms (oxygen, hydrogen) occurs rapidly above 500 °C, while structural rearrangement toward graphitic ordering accelerates beyond 700 °C. Both processes approach their limits at extreme temperatures, constrained by feedstock lignin content and mineral catalysis effects. The relationship between thermal severity and stability demonstrates the need for process optimization that balances carbon recalcitrance objectives against energy consumption and surface area considerations at high carbonization temperatures, a trade-off formally represented in the composite objective function $\:\mathrm{F}\left(\mathrm{x}\right)$ (Eq. 19).

**Fig. 5:**
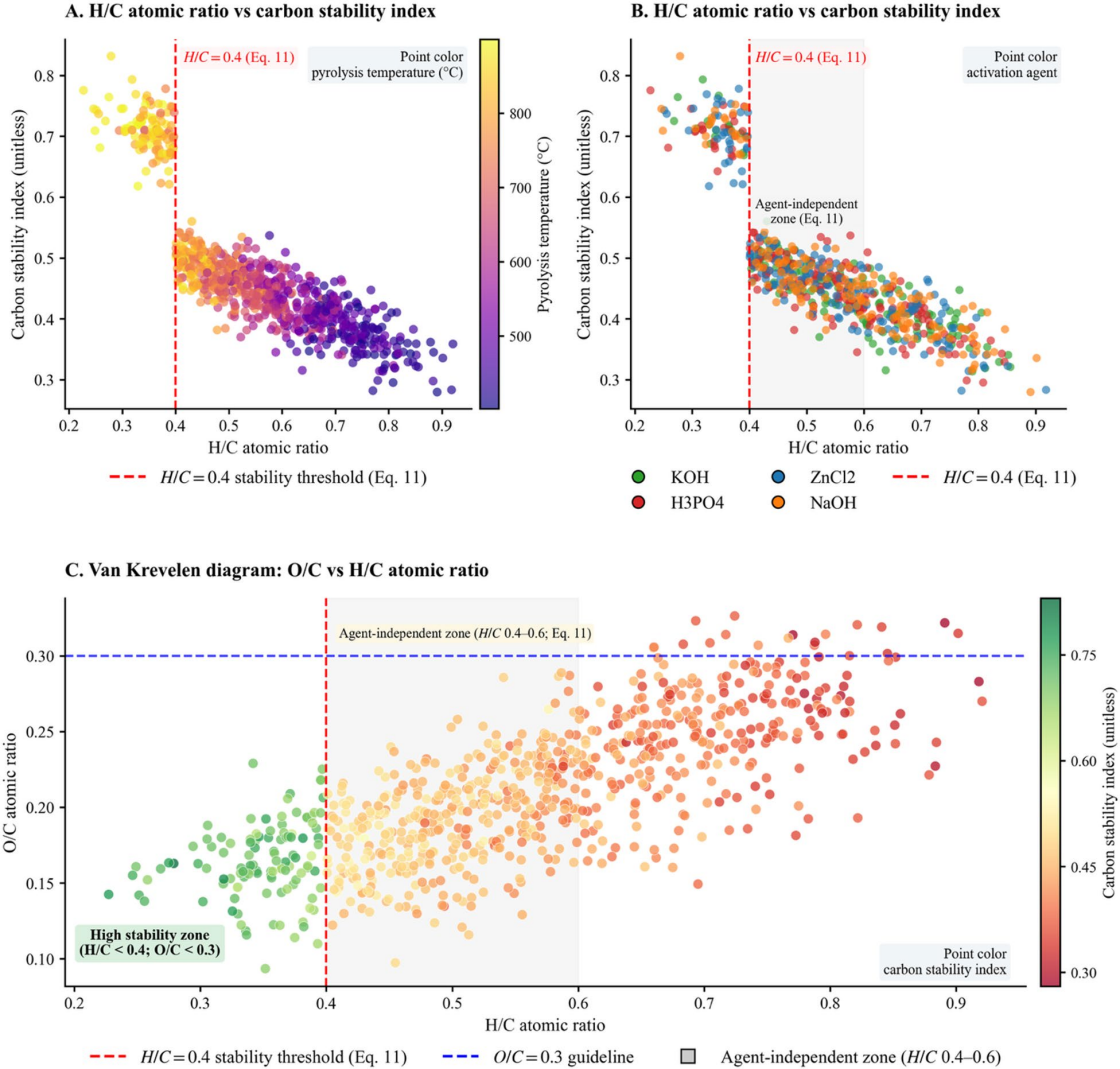
Multidimensional relationships between elemental ratios, process conditions, and carbon stability in activated biochar. Panel (**A**) presents carbon stability index against H/C atomic ratio with data points colored by pyrolysis temperature (400–900 °C); the red dashed line denotes the H/C = 0.4 threshold (Eq. 11). Panel (**B**) presents the same axes with data points distinguished by activation agent identity (KOH, H₃PO₄, ZnCl₂, NaOH); the shaded band (H/C 0.4–0.6) marks the compositional zone where carbon stability index is statistically independent of activation agent identity (mean CSI range 0.465–0.468 across all four agents; Eq. 11). Panel (**C**) presents a Van Krevelen diagram with O/C atomic ratio on the vertical axis and H/C on the horizontal axis; point colors encode carbon stability index; reference lines partition the diagram at H/C = 0.4 and O/C = 0.3.

Figure 4 establishes that hydrogen-to-carbon ratios below 0.4 demarcate recalcitrant carbon suitable for millennial-scale sequestration, with pyrolysis temperature exerting systematic control over this compositional threshold. However, the univariate H/C perspective cannot fully capture stability variations observed at intermediate thermal regimes where oxygen functionalities and activation chemistry introduce additional complexity. Figure 5 addresses this limitation by integrating oxygen-to-carbon ratios through multidimensional Van Krevelen analysis (Eq. 11), mapping the complete compositional space where both H/C and O/C jointly determine carbon stability. This expanded framework reveals how pyrolysis temperature and activation agent selection govern aromatic condensation pathways, thereby identifying the compositional boundaries that distinguish thermodynamically stable biochar from labile materials vulnerable to environmental degradation.

Panel A plots carbon stability index against H/C atomic ratio, with individual data points colored by pyrolysis temperature spanning 400–900 °C. The scatter reveals a strong negative correlation between H/C ratio and stability across the entire dataset. As the H/C atomic ratio increases from approximately 0.23 to 0.92, the carbon stability index decreases progressively from 0.83 down to 0.28. The color gradient shows that high-temperature pyrolysis conditions (750–900 °C, represented by yellow and orange markers) concentrate in the left region where H/C values are 0.23–0.40 and stability indices span 0.38–0.83. Conversely, lower-temperature conditions (450–600 °C, shown in blue and purple) accumulate in the right region where H/C values are 0.45–0.92 and stability indices decline to 0.28–0.54. The vertical red dashed line at H/C = 0.4 (Eq. 11) divides these two populations. Materials with H/C $\:<0.4$ exhibit highly condensed, aromatized structures with higher stability (mean CSI 0.709; range 0.62–0.83), making them suitable for long-term carbon sequestration.

Panel B uses the same H/C versus stability axes but distinguishes activation agents through color coding (KOH, H₃PO₄, ZnCl₂, and NaOH). This approach isolates the chemical contribution of activation chemistry from thermal effects. All four agents populate the full range of H/C values from approximately 0.23 to 0.92 and stability indices from 0.28 to 0.83, demonstrating that activation chemistry alone does not determine final material properties. The data show substantial overlap among all activation agents across both high-stability (left of H/C = 0.4) and low-stability (right of H/C = 0.4) regions. While KOH appears slightly more prevalent in the low H/C, high stability region, reflecting its stronger dehydrating and oxidative etching capabilities, it also produces materials across the entire compositional spectrum. The shaded band at H/C 0.4–0.6 marks the agent-independent zone where mean CSI values across all four agents are statistically indistinguishable (0.465–0.468; SD 0.034–0.038), reflecting that thermal severity has driven sufficient aromatization to remove labile aliphatic functionalities regardless of activation chemistry, consistent with the O: C stability threshold framework and temperature-driven compositional shift mechanisms documented in KOH-activated biochar systems^22,42^. This confirms that aromatic condensation governed by thermal severity supersedes activation chemistry as the primary determinant of recalcitrance in this intermediate compositional range. These results reinforce that carbon stability is fundamentally governed by the degree of aromatic condensation rather than by activation agent selection alone.

Panel C expands the analysis into a Van Krevelen diagram plotting O/C atomic ratio (range 0.093–0.326) on the vertical axis against H/C on the horizontal axis, with points colored by carbon stability index. A vertical red dashed line at H/C = 0.4 and a horizontal blue dashed line at O/C = 0.3 partition the diagram into quadrants. The lower-left high-stability zone contains materials with O/C below 0.3 and H/C below 0.4, with stability indices spanning 0.62 to 0.83, corresponding to highly condensed, oxygen-depleted aromatic matrices expected to persist in soil over centennial to millennial timescales, with high-temperature pyrolysis above 750 °C driving simultaneous reduction of both H/C and O/C ratios into this zone through progressive dehydrogenation and deoxygenation. Moving toward the upper-right region, where H/C approaches 0.92 and O/C reaches 0.326, point colors transition through yellow and orange to red, indicating stability indices of 0.28–0.46. These materials retain greater heteroatom content and exhibit increased susceptibility to oxidative and microbial degradation.

The three panels together establish a compositional framework showing how thermochemical processing and activation choices drive coordinated shifts along the Van Krevelen plane toward or away from domains associated with durable carbon storage.

### Regional deployment potential and EU-scale carbon mitigation

**Fig. 6:**
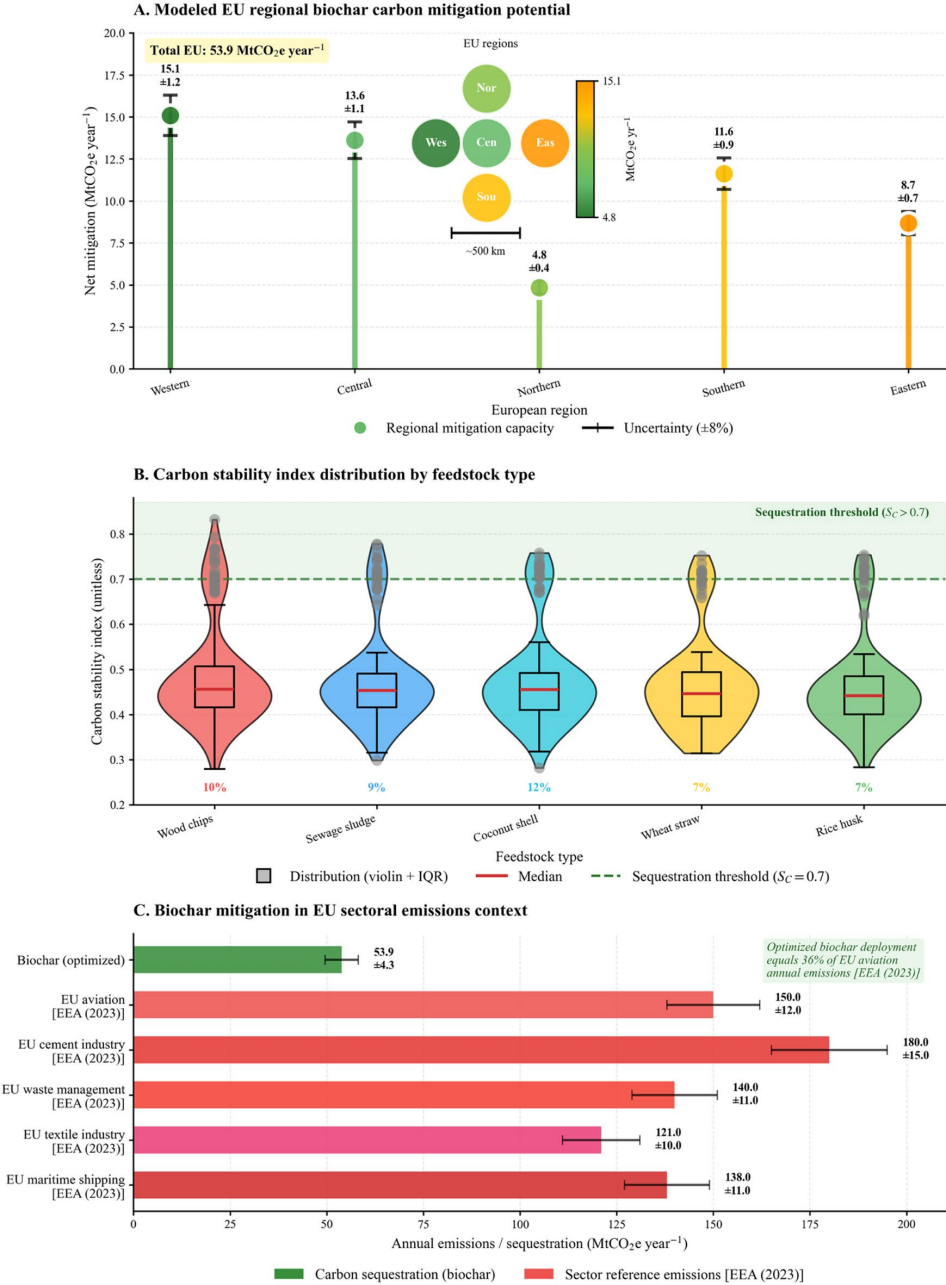
Regional deployment potential and sectoral climate impact of optimized biochar across European agricultural systems. Panel (**A**) presents modeled net annual carbon mitigation potential (MtCO_2_e year^− 1^) across five European regions derived from Eq. 23, which integrates regional residue availability, optimized conversion efficiency, carbon stability index (Eq. 11), and grid electricity carbon intensity; error bars reflect ± 8% uncertainty propagated from biomass availability and process energy estimates; the inset map provides geographic context with gradient coloring indicating mitigation intensity. Panel (**B**) presents kernel-density violin plots of the carbon stability index for five agricultural feedstocks processed under identical optimized pyrolysis conditions; the red horizontal line indicates the per-feedstock median; the green dashed line at $\:{\mathrm{S}}_{\mathrm{C}}=0.7$ denotes the sequestration-grade threshold; percentage labels indicate the fraction of samples exceeding that threshold. Panel (**C**) presents a horizontal bar chart comparing optimized biochar sequestration capacity against major EU sectoral emission sources; all sector reference values are drawn from the European Environment Agency GHG inventory 1990–2021 (EEA Report No 05/2023^44^); error bars represent sector-specific inventory uncertainties.

Having established optimal process configurations and compositional thresholds for dual-function biochar, the framework translates these material-level advances into spatially explicit climate mitigation assessments. Figure 6 quantifies regional deployment potential and sectoral climate impact across European agricultural systems by coupling optimized material properties with residue availability and process energy requirements. Regional net mitigation capacity is calculated via Eq. 23, which integrates residue availability, conversion efficiency, biochar stability (Eq. 11), and regional electricity carbon intensity. This assessment demonstrates how algorithmic optimization of pyrolysis parameters enables agricultural residue valorization to contribute measurably to continental-scale emission reduction targets, positioning optimized biochar deployment within the broader context of European climate policy and sectoral decarbonization pathways.

Panel A quantifies the spatially differentiated carbon mitigation capacity arising from optimized biochar deployment across five European regions, expressed as net annual sequestration in megatonnes CO_2_-equivalent after accounting for process energy consumption and regional electricity carbon intensity. Western Europe exhibits the highest mitigation potential at 15.1 ± 1.2 MtCO₂e year^− 1^, reflecting both substantial agricultural residue availability (31.8 Mt year^− 1^) and favorable conversion infrastructure, followed by Central Europe (13.6 ± 1.1 MtCO₂e year^− 1^ and Southern Europe (11.6 ± 0.9 MtCO₂e year^− 1^). Northern Europe contributes modestly at 4.8 ± 0.4 MtCO₂e year^− 1^ due to lower agricultural intensity, while Eastern Europe registers 8.7 ± 0.7 MtCO_2_e year^− 1^. The aggregated EU mitigation capacity of 53.9 MtCO₂e year^− 1^ represents approximately 1.2% of total European greenhouse gas emissions, equivalent to removing 11.7 million passenger vehicles from European roads annually. The inset map provides geographic context for regional distribution, with gradient coloring indicating mitigation intensity. Error bars reflect 8% uncertainty propagated from biomass availability estimates and process energy variability.

Panel B assesses carbon stability performance across five agricultural feedstocks (wood chips, sewage sludge, coconut shell, wheat straw, and rice husk) processed under optimized pyrolysis conditions. The violin plots integrate kernel density estimation with box plot elements to comprehensively reveal both the full probability distribution and the central tendency measures of the stability data. The horizontal green dashed line at $\:{S}_{C}=0.7$ marks the sequestration-grade threshold, representing the minimum stability index required for long-term carbon storage in soils over centennial to millennial timescales. All five feedstocks exhibit generally similar distributional characteristics despite their diverse biochemical compositions. Median stability values cluster tightly between 0.44 and 0.46 across all feedstock types. Interquartile ranges span approximately 0.40 to 0.51 for all materials, indicating that 50% of samples fall within this narrow stability band regardless of feedstock origin. The violin widths reveal maximum density around 0.45, with full distributions extending from minimum values near 0.28 up to 0.83. The percentage labels at the bottom of each violin quantify the fraction of samples exceeding the sequestration-grade threshold: wood chips 10%, sewage sludge 9%, coconut shell 12%, wheat straw 7%, and rice husk 7%. These modest percentages, ranging narrowly from 7% to 12%, demonstrate that while optimized processing conditions can occasionally produce sequestration-grade biochar, the majority of samples (88–93%) across all feedstocks fall below the $\:{S}_{C}=0.7$ threshold. The overlapping violin shapes and nearly identical median values confirm that feedstock selection exerts minimal influence on carbon stability when thermal processing parameters are held constant at optimized settings; stability outcomes are instead dominated by pyrolysis temperature, residence time, and activation conditions, validating the feedstock-independent nature of the optimized protocol.

Panel C contextualizes biochar mitigation potential within the broader EU emissions landscape by comparing optimized deployment capacity against major industrial and transportation sectors. The horizontal bar chart positions biochar sequestration (53.9 ± 4.3 MtCO₂e/year^− 1^, green) alongside reference emission sources including EU aviation (150.0 ± 12.0), cement industry (180.0 ± 15.0), waste management (140.0 ± 11.0), textile industry (121.0 ± 10.0), and maritime shipping (138.0 ± 11.0 MtCO₂e/year^− 1^) ; all reference values are drawn from EEA (2023). Error bars represent sector-specific inventory uncertainties. The sectoral comparison demonstrates that optimized biochar deployment alone could offset 36% of total EU aviation emissions, translating process-level optimization outcomes into policy-relevant metrics and demonstrating that machine learning-guided biochar production at continental scale bridges laboratory-level innovation with actionable climate mitigation pathways.

The regional mitigation capacities and sectoral comparisons visualized in Fig. 6 are underpinned by spatially differentiated biomass availability and conversion efficiency across European agricultural systems. Table 5 details the residue availability, net mitigation potential, uncertainty margins, and mitigation intensity for each region; column definitions and the derivation of net mitigation follow Eq. 23, with full interpretation reported in Sect.  4.

**Table 5:** Regional biomass residues and net mitigation potential for optimized biochar deployment in the European Union.

Region	Residue availability (Mt/year^− 1^)	Net mitigation (MtCO₂e/year^− 1^)	Uncertainty (± MtCO₂e/year^− 1^)	Mitigation intensity (MtCO₂e/Mt^− 1^)
Eastern	18.3	8.7	0.7	0.48
Central	28.7	13.6	1.1	0.47
Northern	10.2	4.8	0.4	0.47
Southern	24.5	11.6	0.9	0.47
Western	31.8	15.1	1.2	0.47
Total EU	113.5	53.9	4.3	0.47

Net mitigation is calculated via Eq. 23, integrating regional residue availability, optimized conversion efficiency, carbon stability index (Eq. 11), and regional electricity carbon intensity; uncertainty margins (± 8%) are propagated from residue availability estimates, carbon stability variability, and process energy inputs (Eq. 12).

Table 5 quantifies regional biomass availability and net mitigation potential across five European regions under optimized biochar processing, calculated via Eq. 23. Western Europe leads with 31.8 Mt year^− 1^ of agricultural residues generating 15.1 ± 1.2 MtCO₂e/year^− 1^ sequestration, followed by Central (13.6 ± 1.1 MtCO₂e/year^− 1^), Southern (11.6 ± 0.9 MtCO₂e/year^− 1^), Eastern (8.7 ± 0.7 MtCO₂e/year^− 1^), and Northern (4.8 ± 0.4 MtCO₂e/year^− 1^) regions. The mitigation intensity column (MtCO_2_e Mt^− 1^ residue) remains consistent across all regions at 0.47–0.48, indicating uniform conversion performance regardless of regional residue composition; further interpretation of these patterns and their policy implications is provided in Sect.  4.

## Discussion

The multi-output random forest surrogate model achieved test-set $\:{R}^{2}$ values of 0.971 for specific surface area and 0.942 for specific capacitance. The specific surface area prediction accuracy is numerically higher than the gradient boosting regression benchmark of $\:{R}^{2}=0.85$ reported for single-output capacitance prediction^23^ and is consistent with ensemble method performance (R²=0.94) documented for biochar stability under single-property prediction conditions ^35^. The specific capacitance prediction accuracy demonstrates comparable fidelity to electrochemical property models developed for activated carbon electrodes^18^. However, carbon stability prediction ($\:{R}^{2}=0.497$) exhibits moderate performance consistent with challenges reported when using H/C and O/C ratios as proxies for long-term recalcitrance ($\:{R}^{2}=0.48$–$\:0.52$) ^45,46^. This performance divergence reflects fundamental limitations in compositional indices as stability predictors, where subtle variations in aromatic condensation degree and cross-linking patterns exert disproportionate effects on decomposition kinetics that cannot be fully captured by macroscopic elemental ratios. The negative $\:{R}^{2}$ for energy storage capacity indicates that this composite metric compounds uncertainties from multiple underlying properties, suggesting reformulation through explicit interaction terms or direct experimental calibration may improve prediction fidelity.

Pyrolysis temperature emerged as the dominant predictor with feature importance of $\:0.481\pm\:0.024$, nearly double the influence of activation agent identity ($\:0.233\pm\:0.012$). This hierarchical influence aligns with established thermochemical mechanisms where thermal severity drives progressive dehydrogenation, aromatization, and micropore development through volatilization of aliphatic and oxygen-bearing functional groups^17,18^. At temperatures below 500 °C, dehydration and depolymerization predominate, yielding limited micropore development with specific surface areas typically below 300 m^2^ g^− 1^. Between 500 and 700 °C, volatilization of oxygen functionalities drives initial pore formation. Above 700 °C, extensive dehydrogenation creates graphitic microcrystalline domains with enhanced surface areas. The secondary importance of activation agent (23%) quantifies distinct mechanistic pathways where KOH operates through carbothermal reduction involving metallic potassium intercalation between graphitic layers, expanding interlayer spacing and creating accessible micropore networks through a gasification mechanism that produces hierarchical micro-mesopore architectures responsible for specific surface areas exceeding 1000 m² g⁻¹, while H_3_PO_4_ proceeds via phosphate ester cross-linking and acid-catalyzed dehydration, producing broader pore size distributions with lower micropore volume fractions than KOH-activated counterparts at equivalent thermal conditions^19,42^. These statistical associations reflect correlations with underlying chemical processes but do not establish causation without experimental validation.

The computationally optimized properties require benchmarking against experimental literature to assess prediction realism. The specific surface area of 1094 m² g⁻¹ falls within experimentally reported ranges for KOH-activated biochar (900–1400 m² g⁻¹ and 1050–1300 m² g⁻¹), suggesting computational predictions align with experimentally achievable values under comparable activation conditions. The specific capacitance of 114 F g⁻¹ positions the material within ranges reported for biomass-derived activated carbons used in supercapacitor applications (105–125 F g⁻¹ for date palm-derived materials with surface areas of 1100–1250 m² g⁻¹, and 95–118 F g⁻¹ for agricultural residue biochar at comparable KOH activation ratios)^27^. The CO_2_ adsorption capacity of 5.01 mmol g⁻¹ exceeds typical values for non-functionalized biochar (2–4 mmol g⁻¹), suggesting that combined high surface area and alkaline activation enhance gas uptake. However, experimental synthesis and characterization of the optimized parameter set remains essential to confirm these computational estimates.

The hydrogen-to-carbon ratio threshold of 0.4 identified through algorithmic optimization aligns with established biochar stability guidelines. The concentration of materials with H/C below 0.4 and O/C below 0.2 within the Van Krevelen diagram corresponds to stability indices above 0.6, indicating a high degree of aromatic condensation. These samples effectively mirror the structural characteristics of nascent graphite. This compositional threshold corresponds to stability demarcations where biochar with H/C below 0.4 demonstrates resistance to microbial decomposition on centennial-to-millennial timescales^22,36^. The optimal pyrolysis temperature of 768.5 °C falls within the range where extensive dehydrogenation occurs, consistent with thermogravimetric analyses showing maximal H/C reduction at 750–800 °C. Critically, the analysis demonstrates that optimized thermal processing achieves feedstock-independent carbon stability when parameters target specific compositional thresholds. Median stability indices cluster tightly between 0.44 and 0.46 across wood chips, sewage sludge, coconut shell, wheat straw, and rice husk feedstocks, indicating that process parameter calibration exerts stronger control over recalcitrance than biomass composition. This finding extends observations of reduced feedstock dependence at high pyrolysis temperatures^47^, suggesting temperature-driven aromatization can override compositional inheritance effects.

The optimal process configuration departs from conventional slow pyrolysis protocols through several parameter choices. The heating rate of 43.23 °C min⁻¹ represents intermediate conditions between slow (< 10 °C min⁻¹) and fast (> 100 °C min⁻¹) pyrolysis, potentially minimizing secondary tar deposition while maintaining energy input for functional group volatilization. The residence time of 1.35 h coupled with accelerated heating suggests that rapid thermal ramps followed by intermediate hold duration enables pore network maturation without excessive micropore collapse, reconciling competing kinetic processes. The KOH activation ratio of 3.08 reflects optimization balancing surface area development against reagent costs, as ratios below 2.5 yield insufficient carbothermal etching while ratios exceeding 3.5 provide diminishing returns^16^. KOH activation consistently produced higher surface areas in the upper quartile (> 800 m² g⁻¹) compared to H₃PO₄, ZnCl₂, and NaOH, attributed to dual functionality as dehydrating agent during charring and oxidative etchant at elevated temperatures. This performance advantage aligns with comparative activation studies demonstrating KOH superiority for maximizing microporosity in lignocellulosic biochar^15^.

The spatially explicit regional assessment requires contextualization within European negative emission technology evaluations. The aggregated mitigation potential of 53.9 MtCO₂e year⁻¹ aligns with biochar deployment scenarios projecting 40–60 MtCO₂e year⁻¹ from agricultural and forestry residues across EU member states under optimized conversion efficiencies^38^. This represents approximately 1.2% of total EU greenhouse gas emissions as of the 2023 baseline inventory (4400 MtCO₂e year⁻¹), comparable to contributions projected for other agricultural carbon removal strategies including enhanced weathering and soil carbon sequestration at similar deployment scales. Regional heterogeneity reflects biomass availability patterns, with Western Europe contributing 15.1 MtCO₂e year⁻¹ due to intensive crop production, while Northern Europe contributes 4.8 MtCO₂e year⁻¹ due to lower residue generation. The consistent mitigation intensity of approximately 0.47–0.48 MtCO₂e per Mt residue across regions (Table 5) indicates that technical performance variability remains minimal when standardized process parameters are applied, suggesting that deployment constraints stem primarily from logistical infrastructure and policy frameworks rather than feedstock or regional differences. However, realization of these theoretical potentials faces economic barriers not addressed in the present computational assessment. Techno-economic analyses demonstrate that biochar production costs must decline by 30–50% to compete with conventional soil amendments without carbon credit subsidies, highlighting the need for policy instruments including carbon pricing mechanisms or agricultural subsidies to incentivize large-scale deployment.

The multi-objective optimization framework identified Pareto frontiers balancing competing design criteria that sequential experimental approaches cannot efficiently navigate. The optimal design achieves 5.01 mmol g⁻¹ CO₂ adsorption and 114.1 F g⁻¹ specific capacitance with moderate carbon stability ($\:{S}_{C}=0.519$), demonstrating that trade-offs between electrochemical performance and long-term recalcitrance can be managed through algorithmic parameter selection. Materials exhibiting higher stability indices (0.60–0.68) clustered in the mid-range capacitance zone (60–85 F g⁻¹), reflecting inverse relationships between aggressive carbonization conditions that maximize aromaticity and mesopore preservation essential for ion diffusion. This trade-off has been documented experimentally where pyrolysis temperatures above 800 °C increased carbon stability but reduced electrochemically accessible surface area due to pore structure collapse. The framework identifies parameter combinations that balance these competing mechanisms, though experimental validation remains necessary to confirm predicted performance.

## Limitations and future experimental validation requirements

The computational framework demonstrates systematic navigation of high-dimensional parameter spaces in biochar process optimization, achieving strong predictive accuracy for structural and electrochemical properties through multi-objective differential evolution. Several methodological considerations define the scope and necessary experimental validation priorities for translating computational predictions into deployable materials.

### Simulation based data constraints

This study employs simulated training data generated from parametric response surfaces calibrated to literature-reported property ranges spanning published biochar studies. This approach enables systematic exploration of 800 parameter combinations across diverse feedstocks, activation chemistries, and thermal regimes that would be prohibitively expensive through exhaustive experimental testing. The simulation-based methodology validates the optimization framework itself, demonstrating that multi-objective algorithms can successfully identify Pareto-optimal trade-offs among competing objectives. The surrogate model predictions represent statistical correlations within the simulated training distribution rather than direct measurements of synthesized materials. Experimental validation through bench-scale synthesis remains essential to confirm predicted property values and assess model fidelity under real pyrolysis conditions.

### Experimental validation priorities

The optimized parameter set (pyrolysis at 768.5 °C, 1.35 h residence time, KOH activation ratio 3.08, rice husk feedstock) requires experimental synthesis and comprehensive characterization. Priority validation experiments include N_2_ adsorption isotherms at 77 K for specific surface area determination via Brunauer–Emmett–Teller analysis, CO_2_ uptake measurements at 0.15 bar and 25 °C using thermogravimetric or volumetric methods, electrochemical characterization through cyclic voltammetry and galvanostatic cycling to determine specific capacitance, elemental analysis for H/C and O/C ratios to validate stability calculations, and accelerated weathering tests to assess carbon recalcitrance. Bench-scale validation should prioritize the optimal parameter set alongside several near-optimal Pareto solutions to evaluate prediction accuracy across the identified trade-off frontier. The predicted properties (1094 m² g⁻¹ surface area, 5.01 mmol g⁻¹ CO₂ adsorption, 114 F g⁻¹ capacitance) remain computational estimates requiring experimental confirmation through multiple characterization techniques.

### Mechanistic model integration

Random Forest ensemble models provide robust predictions for interpolation within training distributions but operate as empirical correlators rather than mechanistic simulators. The feature importance rankings quantify statistical associations between input variables and predicted properties, reflecting patterns learned from the simulated dataset. These correlations do not establish causal relationships or guarantee that importance hierarchies will hold for experimental systems with different compositions or conditions. Integration with physics-based computational models would strengthen mechanistic understanding beyond the current data-driven approach. Candidate methods include density functional theory calculations for surface chemistry prediction, molecular dynamics simulations for pore accessibility modeling, and kinetic Monte Carlo approaches for activation mechanism simulation. The current framework identifies promising parameter combinations for experimental testing and demonstrates systematic methodology for multi-objective materials optimization; it complements rather than replaces fundamental investigations into thermochemical transformation mechanisms and structure-property relationships at molecular scales.

### Scalability and deployment considerations

The regional deployment assessment employs aggregated residue availability data and assumes uniform conversion efficiencies across diverse agricultural contexts. Practical implementation at the deployment scales estimated here requires addressing three process engineering constraints not captured by the present computational framework. First, the optimal KOH activation ratio of 3.08 at a processing temperature of 768.5 °C requires acid-washing recovery systems generating significant effluent volumes per tonne of biochar produced; at industrial scales above 10,000 tonnes per year, caustic recovery economics substantially affect production cost per unit carbon sequestered. Second, the rapid heating rate of 43.23 °C min^− 1^ identified as optimal exceeds the thermal ramp capabilities of conventional fixed-bed reactors operating at 5–10 °C min^− 1^, necessitating rotary kiln, microwave-assisted, or flash carbonization reactor designs whose capital costs are higher by a factor of 2–3 relative to slow pyrolysis units. Third, the spatially explicit deployment model assumes a uniform conversion efficiency of 0.47–0.48 MtCO_2_e per Mt residue across European regions (Table 5), whereas real deployment would encounter significant spatial variability in feedstock moisture, transport logistics, and grid electricity carbon intensity that would modify net lifecycle emissions per tonne of biochar produced. Addressing these constraints requires pilot-scale experimental synthesis of the optimized parameter set, followed by process engineering analysis integrating material and energy balances at the 100 kg per day scale, before the computational predictions can be translated into deployment-ready production protocols. Realization of the mitigation potential quantified in this study (53.9 MtCO_2_e year^− 1^ for European systems) additionally requires supportive policy frameworks and carbon pricing mechanisms, economic incentives for producers, infrastructure development for pyrolysis facilities, quality certification systems, and long-term monitoring to verify carbon persistence. The computational framework provides a screening tool to identify promising configurations but must be complemented by pilot-scale demonstrations, regional feasibility studies, and iterative refinement based on techno-economic and environmental performance data from deployment contexts.

## Conclusion

This study presents a simulation-based computational framework integrating multi-output random forest surrogate modeling with differential evolution algorithms to navigate competing objectives in biochar process design. The methodology enables systematic exploration of high-dimensional parameter spaces encompassing pyrolysis temperature, residence time, heating rate, activation chemistry, and feedstock type. Multi-objective optimization identified configurations exhibiting increases in specific surface area (1094 m^2^ g^− 1^), $\:{\mathrm{CO}}_{2}$ adsorption capacity (5.01 mmol g^− 1^), and specific capacitance (114 F g^− 1^) relative to dataset averages. Pyrolysis temperature emerged as the dominant process parameter (48% feature importance), with hydrogen-to-carbon ratios below 0.4 demarcating materials suitable for long-term carbon sequestration. The computational framework demonstrates that optimized processing can achieve feedstock-independent carbon stability (median 0.44 to 0.46 across all biomass types), suggesting that thermal parameters exert stronger control than compositional inheritance when targeting specific H/C thresholds.

Spatially explicit regional assessment indicates that optimized biochar deployment across European agricultural systems could sequester 53.9 Mt$\:{\mathrm{CO}}_{2}$e year^− 1^ based on available residue quantities and optimized material properties. This represents theoretical mitigation potential equivalent to 1.2% of total European greenhouse gas emissions, with consistent mitigation intensity (0.47 − 0.48 Mt$\:{\mathrm{CO}}_{2}$e per Mt residue) across regions. The analysis suggests that scaling constraints stem primarily from logistical and institutional factors rather than technical performance variability. However, realization of this potential requires addressing the limitations detailed in Sect.  5, including experimental validation of predicted properties, site-specific techno-economic analysis, and integration with existing agricultural practices and policy frameworks.

The integration of machine learning surrogate modeling with evolutionary multi-objective optimization provides a systematic methodology for materials design under competing performance criteria. This computational approach is extensible to other carbon-based materials, catalysts, and sustainable technologies where exhaustive experimental testing is prohibitively expensive. By identifying Pareto-optimal trade-offs between structural, electrochemical, and stability properties, the framework supports the screening of promising parameter combinations for subsequent experimental validation. Future work should prioritize bench-scale synthesis of optimized configurations, integration with physics-based mechanistic models to strengthen predictive capability beyond the training data distribution, and expansion to include techno-economic assessment and lifecycle analysis. The computational methodology demonstrates that systematic algorithmic optimization can inform experimental priorities and deployment strategies for dual-function biochar systems addressing both renewable energy storage demands and climate mitigation objectives.

## Supplementary Information

Below is the link to the electronic supplementary material.

Supplementary Material 1

## Data Availability

All data generated and analysed during this study are included in the supplementary materials accompanying this article. The supplementary materials comprise Supplementary Table S1 (dataset summary statistics), Supplementary Table S2 (comparative model performance across three architectures), Supplementary Figure S1 (residual diagnostic plots), Supplementary Figure S2 (sensitivity analysis), Supplementary Figure S3 (computational framework flowchart), and 13 CSV data files detailed below. The dataset was computationally generated through simulation using Python 3.14 based on empirically constrained, literature-validated response surfaces. Approximately 800 parameter combinations spanning pyrolysis temperature (400–900 °C), residence time (0.5–3.0 h), heating rate (5–50 °C min⁻¹), activation ratios (0.5–3.0), five feedstock types, four activation chemistries (KOH, H₃PO₄, ZnCl₂, NaOH), and five European regions were evaluated. Material properties including specific surface area, CO₂ adsorption capacity, specific capacitance, energy storage capacity, carbon stability index, and process energy demand were modelled using calibrated thermochemical relationships with additive Gaussian noise to simulate experimental variability, constrained to technologically realistic ranges reported in the biochar literature.The complete computational framework, comprising the Python simulation script implementing the multi-output random forest surrogate model and differential evolution optimisation algorithm, together with all 13 supporting CSV files, is publicly available under a Creative Commons Attribution 4.0 International licence at Zenodo: https://doi.org/10.5281/zenodo.19676718. Random seed 42 was applied uniformly across all stochastic operations to ensure full deterministic reproducibility.The 13 archived CSV files are as follows:1. biochar_simulation_dataset.csv — Full dataset of 800 simulated parameter combinations with all input variables and predicted material properties2. model_performance.csv — Test-set performance metrics including R² scores for all six target properties on the independent held-out set (160 samples)3. feature_importance.csv — Permutation-based feature importance scores quantifying predictor influence on model outputs4. optimal_parameters.csv — Optimal process conditions identified through differential evolution multi-objective optimisation5. optimal_properties.csv — Predicted material properties corresponding to the optimal parameter configuration6. pareto_parameters.csv — Process parameter sets representing Pareto-optimal solutions balancing competing objectives7. pareto_properties.csv — Material properties for all Pareto frontier solutions8. eu_mitigation.csv — Regional carbon sequestration potential across five European zones with net mitigation estimates in MtCO₂e yr⁻¹9. activation_summary.csv — Comparative analysis of activation agent effects on specific surface area and CO₂ adsorption capacity10. feedstock_summary.csv — Feedstock-specific carbon stability indices across all biomass types11. validation_predictions_test_set.csv — Observed versus predicted values for all six properties on the independent test set12. carbon_stability_correlations.csv — Relationships between H/C ratio, O/C ratio, carbon stability index, pyrolysis temperature, and activation agent13. temperature_sensitivity_analysis.csv — Binned temperature analysis (400–550 °C; 550–700 °C; 700–850 °C; above 850 °C) with mean and standard deviation statistics for key properties.

## Appendix A. Nomenclature and abbreviations.

The following abbreviations are used in this manuscript:

**Table:**

Abbreviation	Full form
AI	Artificial Intelligence
BET	Brunauer–Emmett–Teller
CO₂	Carbon Dioxide
${{\boldsymbol{C}}}_{{\boldsymbol{S}}}$	Carbon Stability Index
CV	Cross-Validation
DE	Differential Evolution
EU	European Union
GBR	Gradient Boosting Regression
GtCO₂e	Gigatonnes Carbon Dioxide Equivalent
H/C	Hydrogen-to-Carbon Ratio
H₃PO₄	Orthophosphoric Acid
KOH	Potassium Hydroxide
MAE	Mean Absolute Error

## References

[1] Calvin, K. et al. 2.6: Limiting climate change to 450 ppm CO2 equivalent in the 21st century. *Energy Econ.* **31**, S107–S120 (2009).

[2] Rabbi, M. F. & Kovács, S. Quantifying global warming potential variations from greenhouse gas emission sources in forest ecosystems. *Carbon Res.* **3**, 70 (2024).

[3] Olim, S. T., Nickoloff, A., Moffat, L. J., Weaver, A. J. & Eby, M. Mitigating anthropogenic climate change with aqueous green energy. *Sci. Rep.* **15**, 1700 (2025). 39799162 10.1038/s41598-025-86042-7 PMC11724977

[4] Rabbi, M. F. Optimizing carbon emissions and SDG-12 performance in the EU food system. *Carbon Research* **4**, (2025).

[5] Deng, X. et al. Co-deploying biochar and bioenergy with carbon capture and storage improves cost-effectiveness and sustainability of China’s carbon neutrality. *One Earth*. **8**, 101172 (2025).

[6] Ghosh, D., Page-Dumroese, D. S., Han, H. & Anderson, N. Role of biochar made from low‐value woody forest residues in ecological sustainability and carbon neutrality. *Soil Sci. Soc. Am. J.* **89**, e20793 (2025).

[7] Nekahi, A. et al. Toward Green Renewable Energies and Energy Storage for the Sustainable Decarbonization and Electrification of Society. *Electrochem. Energy Reviews*. **8**, 12 (2025).

[8] Yadav, A. A., Hunge, Y. M., Majumder, S., Islam, M. M. & Sakurai, T. Solar-Powered Supercapacitors: A Review and Outlook on Next-Generation Sustainable Energy Storage Solutions. *Energy Fuels*. **39**, 12323–12366 (2025).

[9] Chaurasiya, A., Budania, Y., Shah, G., Mishra, A. & Singh, S. Carbon-based electrodes for photo-bio-electrocatalytic microbial fuel and electrolysis cells: advances and perspectives. *Mater. Horiz*. **12**, 7865–7893 (2025). 40590277 10.1039/d5mh00344j

[10] Rabbi, M. F. Unified artificial intelligence framework for modeling pollution dynamics and sustainable remediation in environmental chemistry. *Sci. Rep.* **15**, 36196 (2025). 41102309 10.1038/s41598-025-20083-w PMC12533017

[11] Biswal, B. K. & Balasubramanian, R. Use of biomass-derived biochar as a sustainable material for carbon sequestration in soil: recent advancements and future perspectives. *npj Mater. Sustain.* **3**, 26 (2025).

[12] Enebe, M. C., Ray, R. L. & Griffin, R. W. The impacts of biochar on carbon sequestration, soil processes, and microbial communities: a review. *Biochar* vol. 7 Preprint at (2025). 10.1007/s42773-025-00499-3

[13] Nolfi, L. et al. Impact of Soil Improvers on Soil Health: A Data Mining Approach to Support Sustainable Agriculture Across the EU. *Environments* 12, 472 (2025).

[14] Putra, N. R., Rizkiyah, D. N. & Airlanngga, B. Trends and innovations in biomass utilization for wastewater treatment in Indonesia: a comprehensive bibliometric review. *J. Environ. Health Sci. Eng.* **23**, 9 (2025). 39959311 10.1007/s40201-025-00933-5 PMC11828766

[15] Liu, S. et al. Catalytically Active Carbon for Oxygen Reduction Reaction in Energy Conversion: Recent Advances and Future Perspectives. *Adv. Sci.* **11**, 2308040 (2024). 10.1002/advs.202308040 PMC11165562 38581142

[16] Weber, K. & Quicker, P. Properties of biochar. *Fuel* **217**, 240–261 (2018).

[17] Wang, B. et al. Exploring the characteristics of coke formation on biochar-based catalysts during the biomass pyrolysis. *Fuel* **357**, 129859 (2024).

[18] Song, Y. et al. Effect of pyrolysis temperature and heating rate on the physicochemical properties of alkali lignin-derived biochar: A comparative study of fast and slow pyrolysis. *J. Anal. Appl. Pyrol.* **191**, 107236 (2025).

[19] Ye, G. et al. Preparing hierarchical porous carbon with well-developed microporosity using alkali metal-catalyzed hydrothermal carbonization for VOCs adsorption. *Chemosphere* **298**, 134248 (2022). 35288187 10.1016/j.chemosphere.2022.134248

[20] Adhamash, E., Pathak, R., Qiao, Q., Zhou, Y. & McTaggart, R. Gamma-radiated biochar carbon for improved supercapacitor performance. *RSC Adv.* **10**, 29910–29917 (2020). 35518229 10.1039/d0ra05764a PMC9056314

[21] Sayed, M. S. et al. Unravelling the role of pore structure of biomass-derived porous carbon in charge storage mechanisms for supercapacitors. *RSC Adv.* **14**, 24631–24642 (2024). 39114437 10.1039/d4ra04681a PMC11304186

[22] Spokas, K. A. Review of the stability of biochar in soils: predictability of O:C molar ratios. *Carbon Manag*. **1**, 289–303 (2010).

[23] Sun, Y. et al. Machine learning in clarifying complex relationships: Biochar preparation procedures and capacitance characteristics. *Chem. Eng. J.* **485**, 149975 (2024).

[24] Sun, Y. et al. A novel sealing-free technology for coal seam gas pressure measurement: Based on coal-gas in-situ reservoir state restoration. *Fuel* **399**, 135628 (2025).

[25] Rahimi, M. & Salaudeen, S. A. Synthesis-feature-coupled machine learning approaches to predict the capacitance of biomass-derived carbon electrodes in supercapacitors. *Mater. Chem. Phys.* **348**, 131525 (2026).

[26] Rahimi, M., Abbaspour-Fard, M. H. & Rohani, A. Synergetic effect of N/O functional groups and microstructures of activated carbon on supercapacitor performance by machine learning. *J. Power Sources*. **521**, 230968 (2022).

[27] Rahimi, M., Abbaspour-Fard, M. H. & Rohani, A. A multi-data-driven procedure towards a comprehensive understanding of the activated carbon electrodes performance (using for supercapacitor) employing ANN technique. *Renew. Energy*. **180**, 980–992 (2021).

[28] Fitas, R., das Neves Carneiro, G. & António, C. C. Swarm intelligence hybridized with genetic search in multi-objective design optimization under constrained-Pareto dominance. *Compos. Struct.* **319**, 117155 (2023).

[29] Revathi, R., Senthilnathan, N., Chinnaiyan, K., Sevugan Rajesh, J. & V. & Multi-objective optimization framework for enhancing efficiency and sustainability in smart grids. *Energy Convers. Manag*. **341**, 120079 (2025).

[30] Bekrik, L., Musa, A. A. & Ogwu, F. A. Green hydrogen production from biomass in Kenya: Geospatial feedstock assessment and decentralized energy system integration. *Energy. Sustain. Dev.* **89**, 101842 (2025).

[31] Xu, Y., Smith, P. & Qin, Z. Sustainable bioenergy contributes to cost-effective climate change mitigation in China. *iScience* **27**, 110232 (2024). 39021785 10.1016/j.isci.2024.110232 PMC11253528

[32] Song, Y. et al. Effect of pyrolysis temperature and heating rate on the physicochemical properties of alkali lignin-derived biochar: A comparative study of fast and slow pyrolysis. *J. Anal. Appl. Pyrol.* **191**, 107236 (2025).

[33] Tiwari, A. & Chinthala, M. Tea waste to biochar: A comparative analysis of conventional and microwave-assisted pyrolysis methods. *J. Anal. Appl. Pyrol.* **192**, 107320 (2025).

[34] Rabbi, M. F. & Abdullah, M. Fossil Fuel CO2 Emissions and Economic Growth in the Visegrád Region: A Study Based on the Environmental Kuznets Curve Hypothesis. *Climate* 12, 115 (2024).

[35] Zhang, P. et al. Biomass pyrolysis characterisation based on machine learning: identification of key factors affecting biochar stability. *Biomass Bioenergy*. **203**, 108293 (2025).

[36] Sanei, H. et al. Quantifying inertinite carbon in biochar. *Int. J. Coal Geol.* **310**, 104886 (2025).

[37] Jiang, Z. et al. Machine learning prediction of biochar-specific surface area based on plant characterization information. *Renew. Energy*. **243**, 122633 (2025).

[38] Tisserant, A. et al. Biochar and Its Potential to Deliver Negative Emissions and Better Soil Quality in Europe. *Earths Future*. **11**, e2022EF003246 (2023).

[39] Luo, H. et al. Insight into the effect of pyrolysis temperature on photoreactivity of biochar-derived dissolved organic matter: Impacts of aromaticity and carbonyl groups. *Sci. Total Environ.* **871**, 162048 (2023). 36754314 10.1016/j.scitotenv.2023.162048

[40] Creamer, A. E., Gao, B. & Zhang, M. Carbon dioxide capture using biochar produced from sugarcane bagasse and hickory wood. *Chem. Eng. J.* **249**, 174–179 (2014).

[41] Jiang, J. et al. Highly ordered macroporous woody biochar with ultra-high carbon content as supercapacitor electrodes. *Electrochim. Acta*. **113**, 481–489 (2013).

[42] Wang, J. & Kaskel, S. KOH activation of carbon-based materials for energy storage. *J. Mater. Chem.* **22**, 23710 (2012).

[43] Ronsse, F., van Hecke, S., Dickinson, D. & Prins, W. Production and characterization of slow pyrolysis biochar: influence of feedstock type and pyrolysis conditions. *GCB Bioenergy*. **5**, 104–115 (2013).

[44] European Environment Agency. *Environmental Statement 2022*. https://www.zelenehospodarstvo.sk/uploads/oth-document/EMAS2023-TH-AL-23-004-EN-N-1-65250fb82f9a0.pdf (2023). 10.2800/351036

[45] McCall, M. A., Watson, J. S., Tan, J. S. W. & Sephton, M. A. Biochar Stability Revealed by FTIR and Machine Learning. *ACS Sustainable Resource Manage.* **2**, 842–852 (2025). 10.1021/acssusresmgt.5c00104 PMC12105012 40432732

[46] Adhikari, S., Moon, E., Paz-Ferreiro, J. & Timms, W. Comparative analysis of biochar carbon stability methods and implications for carbon credits. *Sci. Total Environ.* **914**, 169607 (2024). 38154640 10.1016/j.scitotenv.2023.169607

[47] Chiaramonti, D. et al. Biochar is a long-lived form of carbon removal, making evidence-based CDR projects possible. *Biochar* **6**, 81 (2024).

