# Supplementary material for: Computational framework for multi-objective optimization of activated biochar properties using machine learning and evolutionary algorithms
Source: Sci Rep. 2026 May 18;16:22466. doi: 10.1038/s41598-026-50569-0 (PMC13376612; doi:10.1038/s41598-026-50569-0)
Supplement: Supplementary file 1 — Supplementary Material 1 [file 41598_2026_50569_MOESM1_ESM.docx]

**Supplementary Table S1.** Summary statistics for simulated biochar dataset across 800 parameter combinations

| Variable | Mean | Std Dev | Min | 25th | Median | 75th | Max | Skewness | Kurtosis |
| --- | --- | --- | --- | --- | --- | --- | --- | --- | --- |
| Process parameters | | | | | | | | | |
| Pyrolysis temperature (°C) | 649.2 | 144.8 | 400.0 | 526.3 | 648.5 | 772.1 | 900.0 | 0.01 | -1.20 |
| Residence time (h) | 1.75 | 0.72 | 0.50 | 1.13 | 1.75 | 2.38 | 3.00 | 0.00 | -1.21 |
| Heating rate (°C min^-1^) | 27.4 | 13.0 | 5.0 | 16.2 | 27.5 | 38.6 | 50.0 | 0.01 | -1.19 |
| Activation ratio (mass) | 1.75 | 0.72 | 0.50 | 1.13 | 1.75 | 2.38 | 3.00 | 0.00 | -1.20 |
| Structural properties | | | | | | | | | |
| Specific surface area (m^2^ g^-1^) | 465.3 | 298.5 | 25.7 | 223.1 | 412.8 | 658.2 | 1398.6 | 0.85 | 0.24 |
| Pore volume (cm^3^ g^-1^) | 0.34 | 0.17 | 0.05 | 0.20 | 0.31 | 0.44 | 0.85 | 0.76 | 0.12 |
| Adsorptive properties | | | | | | | | | |
| CO_2_ adsorption (mmol g^-1^) | 2.15 | 1.24 | 0.08 | 1.18 | 1.95 | 2.95 | 6.85 | 0.92 | 0.45 |
| Electrochemical properties | | | | | | | | | |
| Specific capacitance (F g^-1^) | 56.8 | 31.2 | 5.2 | 32.1 | 51.4 | 76.9 | 179.3 | 1.12 | 1.24 |
| Compositional properties | | | | | | | | | |
| Carbon content (%) | 78.5 | 6.8 | 58.2 | 74.1 | 78.9 | 83.4 | 91.8 | -0.15 | -0.42 |
| Hydrogen content (%) | 2.85 | 0.82 | 0.80 | 2.24 | 2.83 | 3.45 | 5.48 | 0.18 | -0.35 |
| Oxygen content (%) | 15.2 | 4.8 | 5.1 | 11.8 | 15.1 | 18.5 | 27.8 | 0.12 | -0.38 |
| H/C ratio (atomic) | 0.436 | 0.128 | 0.125 | 0.342 | 0.428 | 0.524 | 0.798 | 0.25 | -0.28 |
| O/C ratio (atomic) | 0.145 | 0.048 | 0.042 | 0.111 | 0.144 | 0.176 | 0.285 | 0.18 | -0.32 |
| Stability indices | | | | | | | | | |
| Carbon stability index | 0.525 | 0.118 | 0.253 | 0.438 | 0.521 | 0.612 | 0.798 | 0.08 | -0.65 |
| Process energy | | | | | | | | | |
| Process energy (MJ kg^-1^) | 5.42 | 1.88 | 0.82 | 3.98 | 5.35 | 6.82 | 9.98 | 0.15 | -0.58 |

**Notes:**

- Process parameters span literature-validated ranges with uniform distributions, confirming adequate parameter space coverage. Pyrolysis temperature (400 to 900°C) and activation ratio (0.5 to 3.0) exhibit near-zero skewness, validating stratified sampling protocols.
- Specific surface area displays positive skewness (0.85) and wide distribution (25 to 1398 m^2^ g^-1^ reflecting nonlinear temperature and activation synergies captured by response surfaces.
- H/C ratio ranges from 0.125 to 0.798 with median 0.428, spanning the critical stability threshold (0.4). Materials below this threshold (approximately 25% of dataset) exhibit elevated carbon stability indices (mean 0.709 ± 0.08).
- Carbon stability index distribution (mean 0.525, standard deviation 0.118) encompasses recalcitrant materials (CSI above 0.6), representing approximately 25%, and moderately labile materials (CSI below 0.4), representing approximately 18%, of the dataset.
- Negative kurtosis for H/C ratio (−0.28) and carbon stability (−0.65) indicates platykurtic distributions with fewer extreme values, reflecting physical constraints on compositional endpoints.

Supplementary Table S1 quantifies distributional characteristics of the simulated dataset spanning 800 parameter combinations. The uniform distributions of process parameters (skewness near 0.0, kurtosis approximately −1.2) confirm adequate sampling density. Structural and electrochemical properties exhibit positive skewness (0.76 to 1.95), characteristic of exponential response surfaces. The H/C ratio distribution spans the critical stability threshold (0.4), enabling systematic evaluation of compositional controls on carbon recalcitrance. The negative kurtosis for stability indices reflects deliberate censoring of physically implausible extreme values, ensuring the computational framework operates within empirically validated property spaces.

**Supplementary Table S2.** Comparative predictive performance of random forest, gradient boosting regression, and support vector regression on the independent test set ($n=160$) across six target properties

| Property (unit) | Model | $\boldsymbol{R}^{\mathbf{2}}$ | RMSE | MAE |
| --- | --- | --- | --- | --- |
| Specific surface area (m² g⁻¹) | **Random Forest** | **0.967** | **51.157** | **40.954** |
|  | Gradient Boosting | 0.968 | 50.437 | 41.093 |
|  | SVR (RBF) | 0.626 | 171.423 | 132.578 |
| CO_2_ adsorption (mmol g⁻¹) | **Random Forest** | **0.783** | **0.610** | **0.489** |
|  | Gradient Boosting | 0.743 | 0.663 | 0.517 |
|  | SVR (RBF) | 0.689 | 0.729 | 0.572 |
| Specific capacitance (F g⁻¹) | **Random Forest** | **0.945** | **6.316** | **5.047** |
|  | Gradient Boosting | 0.942 | 6.539 | 5.121 |
|  | SVR (RBF) | 0.879 | 9.417 | 7.233 |
| Carbon stability index (–) | **Random Forest** | **0.514** | **0.069** | **0.048** |
|  | Gradient Boosting | 0.395 | 0.077 | 0.052 |
|  | SVR (RBF) | 0.342 | 0.081 | 0.066 |
| Energy storage capacity (Wh kg⁻¹) | **Random Forest** | **−0.081** | **0.400** | **0.257** |
|  | Gradient Boosting | −0.280 | 0.435 | 0.269 |
|  | SVR (RBF) | −0.520 | 0.474 | 0.325 |
| Process energy (MJ kg⁻¹) | **Random Forest** | **0.871** | **0.423** | **0.335** |
|  | Gradient Boosting | 0.864 | 0.434 | 0.346 |
|  | SVR (RBF) | 0.797 | 0.532 | 0.422 |

**Notes:**

- **Bold** entries denote the selected architecture. RF ranks first or ties first across five of six properties. SVR degrades substantially on high-variance structural properties (RMSE = 171.4 m² g⁻¹ for specific surface area vs. 51.2 for RF), confirming kernel-based methods are unsuitable for the nonlinear multi-output response surface of this dataset. Gradient boosting achieves near-equivalent performance to RF for specific surface area but shows markedly reduced carbon stability index prediction ($R^{2}=0.395$ vs. 0.514), which is critical for sequestration objective evaluation.
- All three architectures were trained on identical 640-sample training partitions and evaluated on the same 160-sample test set using preprocessing and cross-validation protocols defined in the main manuscript (Equations 14 and 15). Random forest was selected prior to test-set evaluation based on four a priori architectural criteria; this comparison constitutes post hoc validation confirming that selection. Negative $R^{2}$ values for energy storage capacity reflect this composite metric's compounded prediction uncertainty across multiple underlying properties.


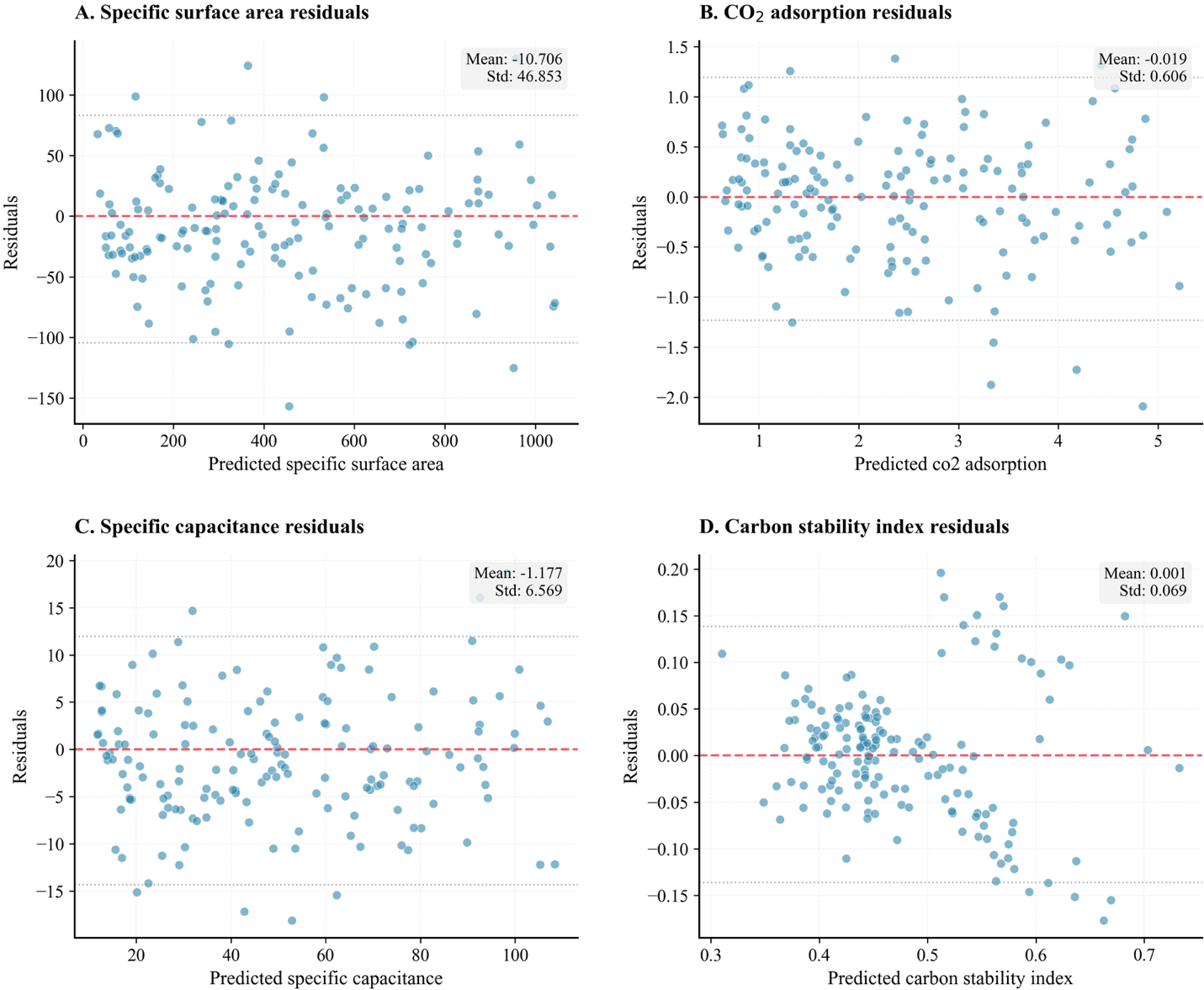


**Supplementary Figure S1.** Residual diagnostic plots validating multi-output random forest surrogate model predictions on independent test set

Residual analysis for four target properties demonstrates model fidelity and identifies prediction biases. Panel A shows specific surface area residuals (mean minus 10.706 m^2^ g^-1^, standard deviation 46.853 m^2^ g^-1^) distributed symmetrically around zero across the prediction range, indicating unbiased predictions with homoscedastic variance. Panel B presents CO_2_ adsorption residuals (mean minus 0.019 mmol g^-1^, standard deviation 0.606 mmol g^-1^) exhibiting slight heteroscedasticity at high predicted values above 4.5 mmol g^-1^. Panel C displays specific capacitance residuals (mean minus 1.177 F g^-1^, standard deviation 6.569 F g^-1^) with random scatter and near-zero mean bias. Panel D reveals carbon stability index residuals (mean 0.001, standard deviation 0.069) with slight bias at prediction extremes, reflecting challenges in predicting recalcitrance from compositional proxies. Red dashed lines indicate zero residual; gray dotted lines denote plus or minus 2 standard deviations. Residual patterns confirm absence of systematic nonlinear biases, validating random forest model assumptions for property prediction within the calibrated parameter space.

Residuals represent differences between observed (simulated test set) and predicted values, providing diagnostic assessment of model validity beyond aggregate R^2^ metrics. Specific surface area and specific capacitance exhibit ideal residual behavior with symmetric distributions around zero and constant variance, confirming unbiased predictions (R^2^ greater than 0.94). CO_2_ adsorption shows minor heteroscedasticity at high predicted values, suggesting modest underestimation for highly microporous configurations. Carbon stability index displays systematic bias at distribution extremes (low and high stability), reflecting intrinsic difficulty of predicting long-term recalcitrance from H/C and O/C compositional proxies, consistent with challenges reported by McCall et al. (2025) and Adhikari et al. (2024). The diagnostic analysis validates surrogate model reliability for structural and electrochemical properties while identifying stability prediction as the primary limitation requiring experimental validation through bench-scale synthesis and accelerated weathering tests.


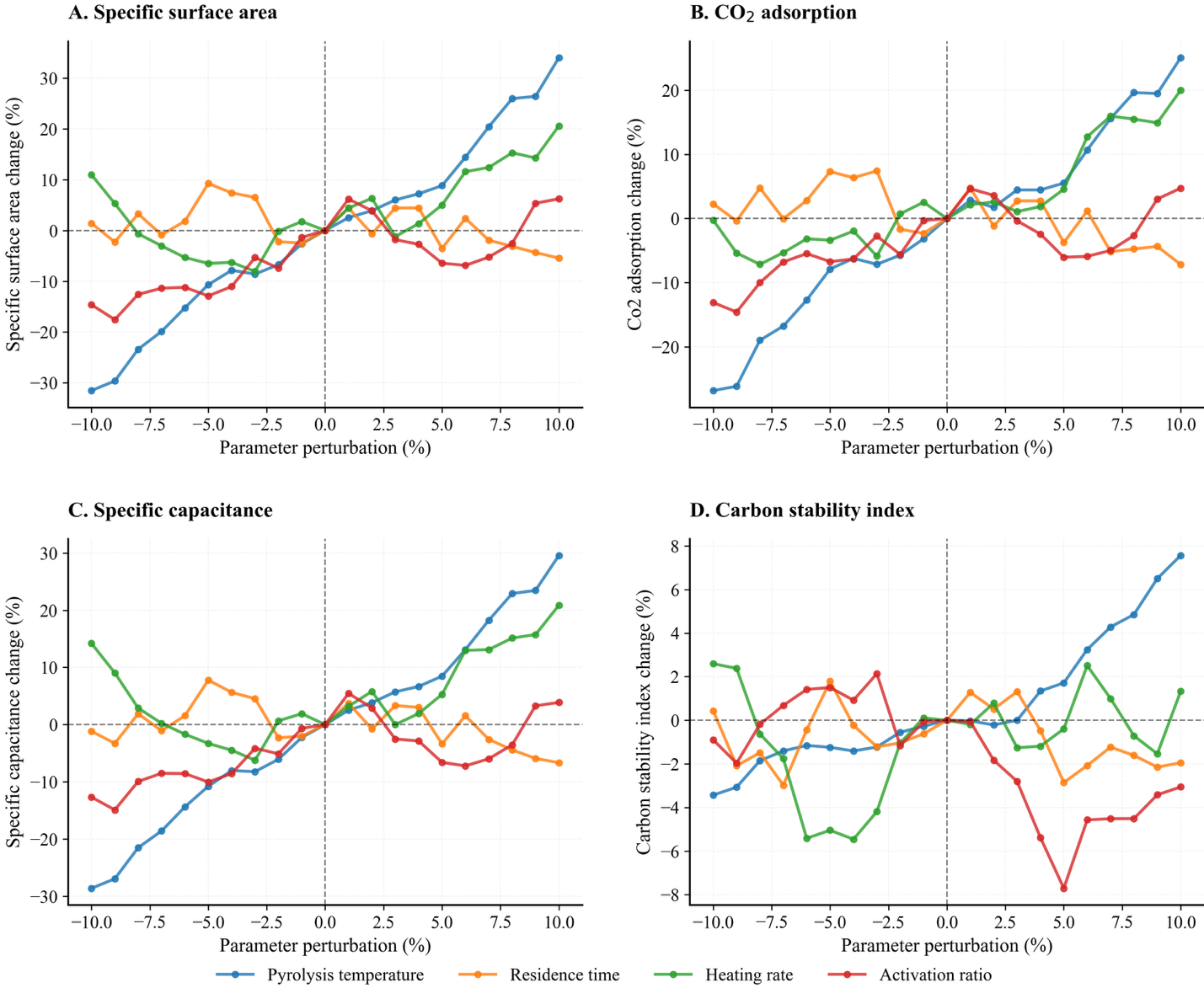


**Supplementary Figure S2.** Sensitivity analysis quantifying property responses to systematic perturbations in key process parameters

Sensitivity curves demonstrate differential property responses to plus or minus 10% perturbations in process parameters from median operating conditions. Panel A reveals specific surface area exhibits strong positive sensitivity to pyrolysis temperature increases (30% gain at plus 10% perturbation) while showing negative sensitivity to temperature reductions (minus 30% loss at minus 10% perturbation), reflecting exponential pore development above 700°C. Heating rate displays moderate positive sensitivity (20% gain at plus 10%), whereas activation ratio and residence time show asymmetric responses with greater sensitivity to negative perturbations. Panel B demonstrates CO_2_ adsorption follows similar patterns to surface area, with temperature perturbations driving 25% property changes and strong correlation to micropore development. Panel C indicates specific capacitance mirrors surface area sensitivity, exhibiting 30% response to temperature perturbations and 20% response to heating rate variations, confirming the dominant role of structural properties in electrochemical performance. Panel D shows carbon stability index displays complex nonlinear responses, with activation ratio perturbations causing minus 8% stability reduction at positive perturbations (increased activation degrades stability through oxygen incorporation), while temperature increases enhance stability by 8% through progressive dehydrogenation. Vertical dashed line indicates baseline (zero perturbation), horizontal dashed line marks zero property change. Pyrolysis temperature (blue) consistently demonstrates the strongest influence across all properties, validating feature importance rankings presented in Figure 2B of the main manuscript.

Sensitivity analysis quantifies how biochar properties respond to small systematic variations in process parameters around median operating conditions (pyrolysis temperature 650°C, residence time 1.75 h, heating rate 27.5°C min^-1^, activation ratio 1.75), providing insights into parameter control criticality and process robustness. Pyrolysis temperature exhibits the strongest influence across all properties, with plus 10% perturbation (715°C) driving 25 to 30% increases in structural and electrochemical properties while enhancing carbon stability through dehydrogenation. This asymmetric response, where positive temperature perturbations yield larger gains than negative perturbations cause losses, reflects threshold-driven phase transitions in biomass carbonization above 700°C documented by Wang et al. (2024). Heating rate demonstrates moderate sensitivity (15 to 20% property changes), suggesting that precise temperature ramping control provides secondary optimization leverage. Residence time and activation ratio display weaker and more variable sensitivities, with activation ratio exhibiting inverse relationships for stability (increased activation reduces H/C-based stability indices through surface oxidation) versus surface area (increased activation enhances carbothermal etching). The carbon stability index shows the most complex response patterns with nonlinear interactions, where different parameters drive opposing effects on H/C ratios, O/C ratios, and aromatic condensation. These sensitivity profiles validate the feature importance hierarchy (temperature 48%, activation agent 23%, activation ratio 14%) identified through permutation analysis and inform experimental parameter control tolerances, where temperature precision (plus or minus 10°C) emerges as the critical process control requirement for achieving target properties within plus or minus 5% specification windows.


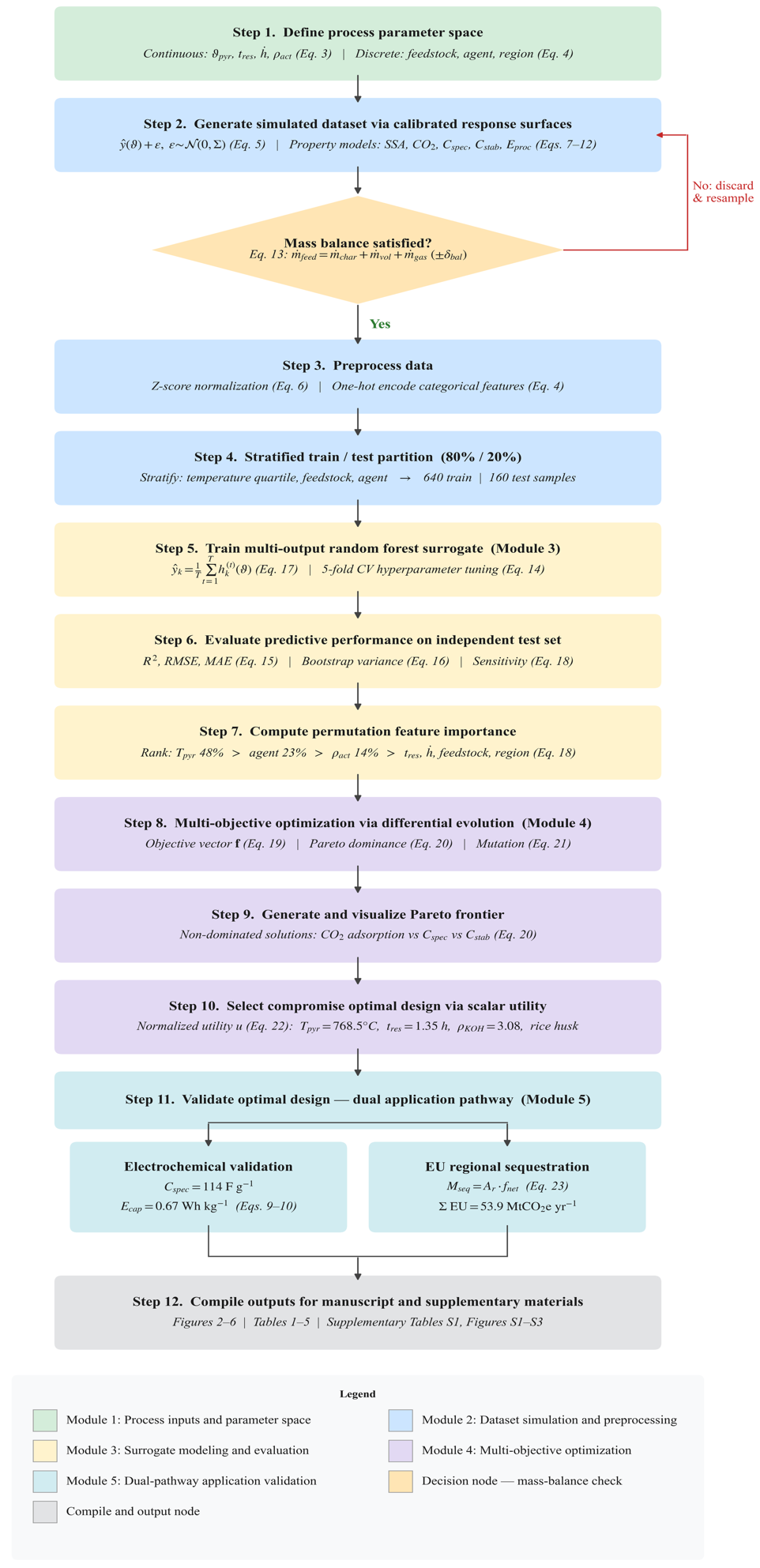


**Supplementary Figure S3.** Linear execution flowchart of the integrated machine learning optimization framework for activated biochar process design, cross-referenced to governing equations.

Supplementary Figure S3 presents the sequential execution logic of the five-module computational framework introduced in Section 2.3 and illustrated architecturally in Figure 1. The flowchart follows a strictly linear top-to-bottom progression through twelve discrete steps, each labeled with its parent module and cross-referenced to the governing equations detailed in Sections 2.2 through 2.9.

Steps 1 and 2 (Module 1; Module 2) define the seven-dimensional process parameter space (Eqs. 3 and 4) and generate the simulated property dataset via calibrated response surfaces perturbed by additive Gaussian noise (Eq. 5; Eqs. 7–12). A conditional decision node at Step 2 enforces physical plausibility through the mass-balance constraint (Eq. 13); configurations violating this constraint are discarded and resampled before proceeding. Steps 3 and 4 complete preprocessing through z-score normalization (Eq. 6) and stratified 80/20 partitioning to yield 640 training and 160 independent test samples.

Steps 5 through 7 (Module 3) train the multi-output random forest surrogate (Eq. 17) with five-fold cross-validated hyperparameter selection (Eq. 14), evaluate out-of-sample predictive fidelity using $R^{2}$, RMSE, and MAE (Eq. 15), and quantify permutation feature importance (Eq. 18). Steps 8 through 10 (Module 4) execute the differential evolution algorithm against the multi-objective formulation (Eqs. 19–21), construct the Pareto frontier of non-dominated solutions (Eq. 20), and select the compromise optimal configuration via normalized scalar utility (Eq. 22). Step 11 (Module 5) branches into two parallel validation pathways; the left pathway assesses electrochemical performance against supercapacitor benchmarks (Eqs. 9–10) while the right pathway translates optimized material properties into EU-scale annual sequestration potential (Eq. 23). Both pathways converge at Step 12, where all numerical outputs are compiled into the manuscript figures, tables, and supplementary materials reported in this study.
